# The role of the tryptophan-NAD + pathway in a mouse model of severe malnutrition induced liver dysfunction

**DOI:** 10.1038/s41467-022-35317-y

**Published:** 2022-12-08

**Authors:** Guanlan Hu, Catriona Ling, Lijun Chi, Mehakpreet K. Thind, Samuel Furse, Albert Koulman, Jonathan R. Swann, Dorothy Lee, Marjolein M. Calon, Celine Bourdon, Christian J. Versloot, Barbara M. Bakker, Gerard Bryan Gonzales, Peter K. Kim, Robert H. J. Bandsma

**Affiliations:** 1 Department of Nutritional Sciences, Temerty Faculty of Medicine, University of Toronto, M5G 1A8 Toronto, Canada; 2 Translational Medicine Program, The Hospital for Sick Children, M5G 0A4 Toronto, Canada; 3 Core Metabolomics and Lipidomics Laboratory, Wellcome Trust-Metabolic Research Laboratories, Institute of Metabolic Sciences, University of Cambridge, CB2 0QQ Cambridge, UK; 4 Biological Chemistry Group, Royal Botanic Gardens, Kew, Kew Green, TW9 3AE Richmond, UK; 5 School of Human Development and Health, Faculty of Medicine, University of Southampton, SO16 6YD Southampton, UK; 6 Department of Metabolism, Digestion and Reproduction, Faculty of Medicine, Imperial College London, SW7 2AZ London, UK; 7 The Childhood Acute Illness & Nutrition Network (CHAIN), Nairobi, Kenya; 8 Laboratory of Pediatrics, Center for Liver, Digestive, and Metabolic Diseases, University of Groningen, University Medical Center Groningen, Groningen, The Netherlands; 9 Nutrition, Metabolism and Genomics Group, Division of Human Nutrition and Health, Wageningen University, Wageningen, The Netherlands; 10 Department of Biochemistry, University of Toronto, M5S 1A8 Toronto, Canada; 11 Cell Biology Program, The Hospital for Sick Children, M5G 0A4 Toronto, Canada; 12 Division of Gastroenterology, Hepatology, and Nutrition, The Hospital for Sick Children, M5G 0A4 Toronto, Canada

**Keywords:** Energy metabolism, Malnutrition, Liver diseases

## Abstract

Mortality in children with severe malnutrition is strongly related to signs of metabolic dysfunction, such as hypoglycemia. Lower circulating tryptophan levels in children with severe malnutrition suggest a possible disturbance in the tryptophan-nicotinamide adenine dinucleotide (TRP-NAD+) pathway and subsequently in NAD+  dependent metabolism regulator sirtuin1 (SIRT1). Here we show that severe malnutrition in weanling mice, induced by 2-weeks of low protein diet feeding from weaning, leads to an impaired TRP-NAD+  pathway with decreased NAD+ levels and affects hepatic mitochondrial turnover and function. We demonstrate that stimulating the TRP-NAD+  pathway with NAD+  precursors improves hepatic mitochondrial and overall metabolic function through SIRT1 modulation. Activating SIRT1 is sufficient to induce improvement in metabolic functions. Our findings indicate that modulating the TRP-NAD+  pathway can improve liver metabolic function in a mouse model of severe malnutrition. These results could lead to the development of new interventions for children with severe malnutrition.

## Abstract (web summary)

Impaired liver metabolic function is related to mortality in severely malnourished children. Here the authors report a role for the tryptophan-NAD + pathway in reduced hepatic mitochondrial function and liver steatosis in a mouse model of severe malnutrition.

## Introduction

Malnutrition contributes to nearly 45% of deaths among children under 5 years of age worldwide^1^. Malnourished children, especially those with severe malnutrition, are at a substantially increased risk of mortality compared to well-nourished children^2^. The current treatment guidelines developed by the World Health Organization (WHO) for children with severe malnutrition are based on limited scientific evidence^3^. Thus, new evidence-based interventions are urgently needed.

The liver is a central organ that regulates nutrient metabolism. In severe malnutrition, hepatic metabolism has been found to be disturbed and is associated with hypoglycemia, hypoalbuminemia, and steatosis^4–6^. Children with severe malnutrition have impaired hepatic glucose production, which increases the risk of hypoglycemia and is related to mortality^5^. We recently discovered in both patients and a rodent model of severe malnutrition, that hepatic mitochondrial function is impaired leading to reduced nutrient oxidation and adenosine triphosphate (ATP) depletion^5,6^. However, the pathophysiology of hepatic mitochondrial dysfunction in severe malnutrition remains poorly understood.

Children with severe malnutrition have been found to have significantly lower serum tryptophan (TRP) levels^7–9^. As an essential amino acid, tryptophan is crucial for growth and protein synthesis. It is also a precursor of nicotinamide adenine dinucleotide (NAD+) and nicotinamide adenine dinucleotide phosphate (NADP+), which are essential co-factors in metabolic and biosynthesis pathways. It has been previously shown that higher urinary excretion of N-methylnicotinamide, a urinary biomarker of NAD+ and nicotinamide availability, was associated with catch-up growth in stunted children^10^. A previous study in low-protein-fed mice also reported changes in the nicotinamide pathway^11^. NAD+ is also a co-substrate for sirtuin1 (SIRT1), which is an important enzyme for mitochondrial health and biogenesis through activation of peroxisome proliferator-activated receptor-gamma coactivator-1 alpha (PGC-1α)^12^. SIRT1 has also been shown to regulate autophagy^13–15^. There have been reports that targeting this pathway in non-alcoholic fatty liver disease (NAFLD) has beneficial effects on hepatic metabolism^16–19^. The role of the TRP-NAD+ pathway in severe malnutrition-associated hepatic metabolic dysfunction remains unknown.

In this study, we aimed to characterize the role of the TRP-NAD+ pathway in hepatic metabolic dysfunction in a mouse model of severe malnutrition. We demonstrate that the TRP-NAD+ pathway is affected in this model and that hepatic mitochondrial dysfunction is related to deficiencies in the TRP-NAD + pathway. We demonstrate supplementing with nicotinamide (NAM) and related components of this pathway improve mitochondrial and overall hepatic metabolic dysfunction. We find that the effects of modulating the TRP-NAD+ pathway are mediated through SIRT1. These findings identify the importance of the TRP-NAD+ pathway and SIRT1 in malnutrition-associated hepatic metabolic dysfunction.

## Results

### Feeding a low-protein diet leads to hepatic steatosis in young mice

**Fig. 1:**
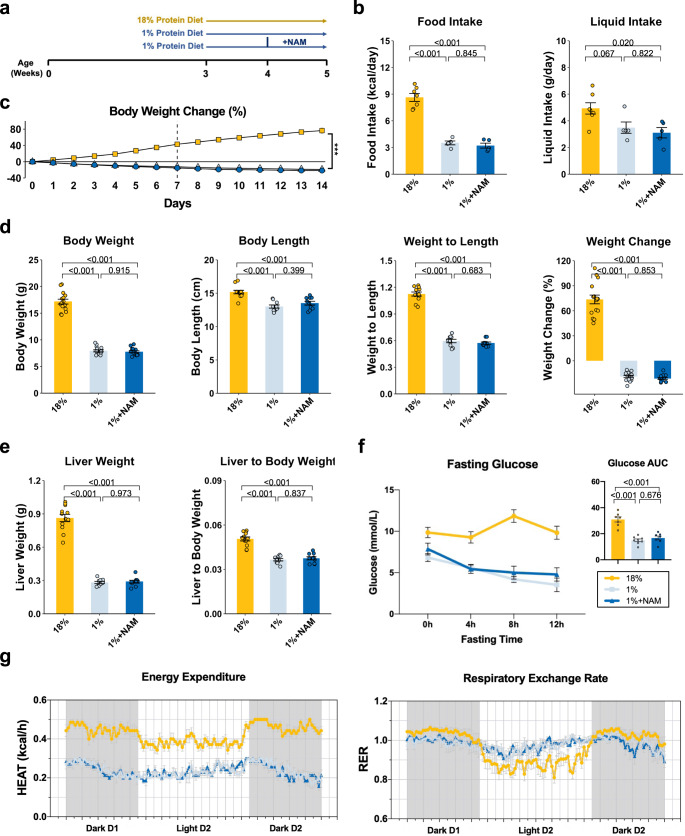
Feeding a 1% protein diet with or without NAM supplementation on basic animal characteristics. **a** Experimental design. **b** Average food and liquid intake from day 7 to day 14 (*n* = 7 mice for 18%; *n* = 5 for 1 and 1%+NAM). **c** Body weight change throughout the experiment (*n* = 15 per group). **d** Final body weight, body length, weight-to-length ratio, weight change assessed on day 14 (*n* = 15 per group). **e** Liver weight and liver weight to body weight ratio (*n* = 12 for 18%; *n* = 10 for 1 and 1%+NAM). **f** Fasting glucose levels (*n* = 7 for 18%; *n* = 8 for 1%; *n* = 7 for 1%+NAM). AUC: area under the curve. **g** Energy expenditure and respiratory exchange rate (RER) of the entire animal. (*n* = 7 for 18%; *n* = 6 for 1%; *n* = 7 for 1%+NAM). One-way ANOVA followed by Tukey’s post hoc test. Data are shown as the mean ± SEM.

To producet a mouse model of severe malnutrition, we fed 3-weeks-old weanling male C57BL/6J mice a 1% protein isocaloric diet for 2 weeks (malnourished group) and compared it to the control group fed an 18% protein diet (control group) (Fig. 1a). Mice subjected to the 1% protein diet lost a significant amount of body weight (approximately 20%) over 2 weeks and had a lower body length and weight-to-length ratio compared to the 18% protein-fed control group (Fig. 1b–d). The 1% protein-fed mice showed a lower liver weight and liver-to-body weight ratio compared to the control (Fig. 1e). Lower fasting glucose and serum albumin levels were also noted in the 1% protein-fed mice (Fig. 1f and Supplementary Fig. 5a), consistent with reduced hepatic glucose and albumin production. As expected, the overall energy expenditure of 1% protein-fed mice was lower than 18% protein-fed controls (Fig. 1g). These differences largely relate to body size, but changes in body composition and/or metabolism could also underlie this finding, especially since we did not find striking differences in locomotor activity (Supplementary Fig. 5b). 1% protein-fed animals showed a clear loss of their day–night circadian pattern in energy expenditure that the normal animals exhibit, which was also reflected by a loss in diurnal variation in the respiratory exchange rate (Fig. 1g).

**Fig. 2:**
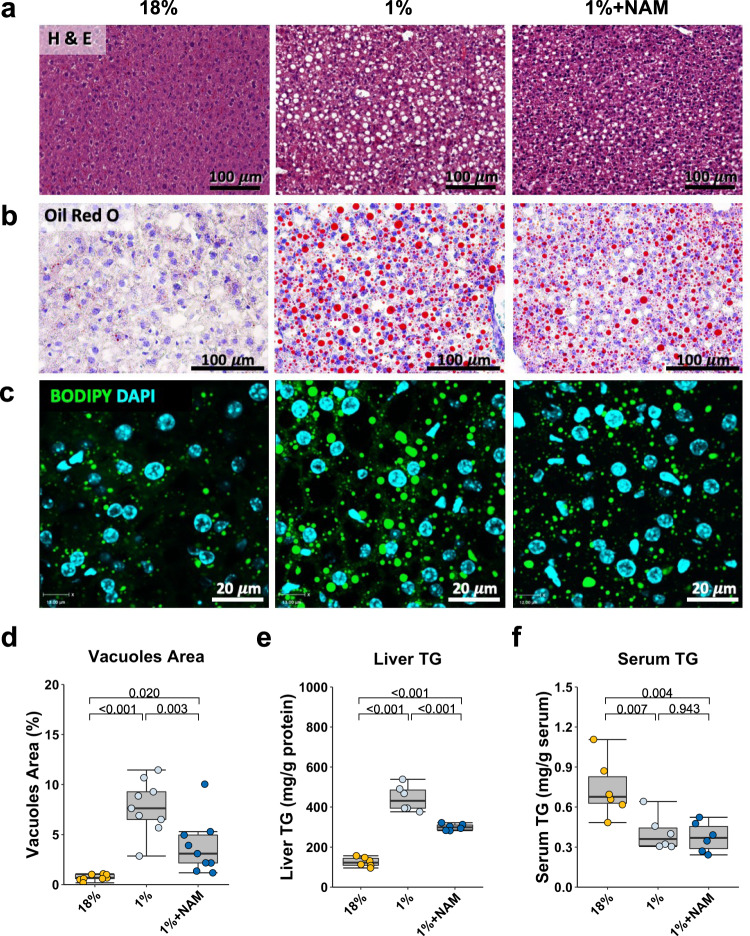
The effect of 1% protein feeding with or without NAM supplementation on hepatic lipid accumulation. **a** Representative hematoxylin and eosin staining images of the liver (×20 magnification). Cytoplasm was stained in red, and the nucleus was stained in purple (*n* = 5 replicates). **b** Representative oil red o stain staining images of the liver (×20 magnification). Fat droplet was stained in red, and nucleus was stained in purple (*n* = 3 replicates). **c** Representative immunofluorescence images of the liver (×40 magnification). BODIPY was used to stain fat droplet in green, and DAPI was used to counter stain nucleus in blue (*n* = 3 replicates). **d** Quantification of vacuoles area (*n* = 9 per group). **e** Liver triglyceride (TG) concentrations (*n* = 6 per group). **f** Serum TG concentrations (*n* = 6 per group). One-way ANOVA followed by Tukey’s post hoc test. Unless stated differently in the figure legend, all n represents biologically independent samples. Data in box plots are shown as the first to third quartile, whiskers encompass the range, and horizontal lines represent the mean. Scale bars are as indicated.

H&E and Oil Red O staining of liver sections identified steatosis in the mice fed with a 1% protein diet as evidenced by an increase in vacuoles and larger fat droplets compared to mice fed with the 18% protein diet (Fig. 2a, b). Both quantifications of vacuole size and visualization of lipid droplet staining with BODIPY supported these results (Fig. 2c, d), which were further validated by measurement of liver triglyceride (TG) levels (Fig. 2e). Serum TGs were lower in the 1% protein-fed group, indicating steatosis is not linked to hypertriglyceridemia (Fig. 2f). Together, these results indicate that the 1% protein diet induces hepatic steatosis in mice similar to those observed in patients and rat models of severe malnutrition^2,6^.

### NAM and TRP-NAD+ pathway modulators reduce the development of low-protein diet-induced hepatic steatosis

Examination for blood tryptophan levels showed the 1% protein-diet mice to be lower than 18% protein-diet control animals (43.0 ± 5.0 μmol/L and 88.4 ± 13.2 μmol/L respectively, *P* = 0.004). To examine the role of reduced tryptophan levels and possible NAM deficiency on liver health, the 1% protein-fed mice were supplemented with 160 mg/kg body weight/day NAM from day 7 to day 14 (Fig. 1a). NAM treatment did not alter the average body weight, body length, or food and liquid consumption in the 1% protein-fed group (Fig. 1b–d). The mice treated with NAM had no significant difference in liver weight, liver/body weight ratio, or fasting glucose levels compared to the untreated 1% protein-diet-fed mice (Fig. 1e, f). Energy expenditure was not affected by NAM treatment (Fig. 1g). NAM treatment improved the hepatic steatosis compared to the 1% protein-fed mice, indicated by a reduction in the vacuoles area and a 30% reduction in liver TG levels compared to untreated animals (Fig. 2a–e). The NAM treatment had no effect on serum TG concentrations (Fig. 2f).

To determine whether the effect of NAM treatment was due to the improvement of the NAD salvage pathway specifically, we treated the 1% protein-fed mice with nicotinamide riboside (NR) or TRP. Both NR and TRP act as NAD+ precursors in the NAD salvage pathway^17^. The allocated interventions were given from day 7 to day 14 (Supplementary Fig. 1a). NR and TRP supplementation, similar to NAM treatment, did not recover body weight, body length or liver weight/body weight ratio compared to the untreated 1% protein-fed group (Supplementary Fig. 1b–e). Similar to the NAM-treated malnourished mice, hepatic steatosis was reduced in the NR- and TRP-treated groups (Supplementary Fig. 2a–f). To determine whether the effects were specific to the TRP-NAD+ pathway, we also performed similar experiments in mice who received supplementation with methionine (MET), another essential amino acid like tryptophan (Supplementary Fig. 1a). This particular amino acid was chosen as MET has been shown to decrease hepatic steatosis in mice on ketogenic diets^20^, and diets completely devoid of MET and choline can induce hepatic steatosis^21,22^. MET supplementation also did not recover body weight and body length, but increased liver weight and body weight ratio compared to the untreated 1% protein-fed mice alone (Supplementary Fig. 1b–e). Supplementation with methionine did not improve hepatic steatosis among the 1% protein-fed mice (Supplementary Fig. 2a–f). Together, these results indicate that supplementation of different NAD+ precursors improves low-protein-induced hepatic steatosis.

### NAM improves low-protein-diet-induced mitochondrial changes

**Fig. 3:**
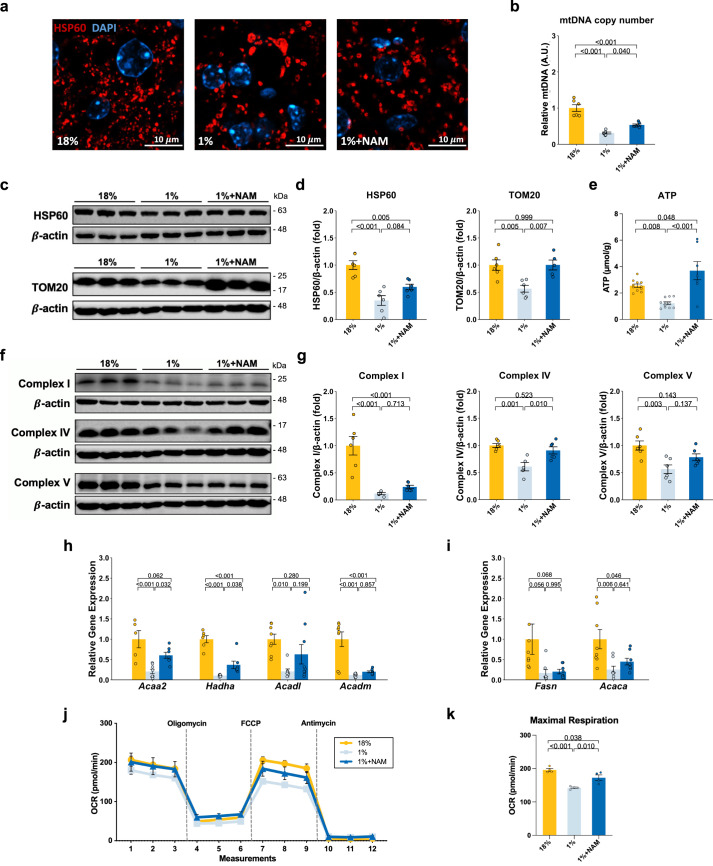
The effect of NAM supplementation on mitochondrial characteristics of 1% protein-fed model. **a** Representative immunofluorescence images of mitochondrial (×60 magnification). HSP60 was used to stain mitochondrial in red, and DAPI was used to counter stain nucleus in blue (*n* = 3 replicates). **b** Relative mtDNA copy number (mtDNA/beta-globin) (*n* = 6 per group). **c**, **d** Representative western blots and quantification of HSP60 and TOM20 (*n* = 3 per group). **e** ATP levels (*n* = 11 for 18 and 1%; *n* = 7 for 1%+NAM). **f, g** Representative western blots and quantification of complex I, complex IV and complex V (*n* = 3 per group). **h** Relative expression of β-oxidation genes compared to control (*n* = 8 for 18%, *n* = 8 for 1%, and *n* = 9 for 1%+NAM). **i** Relative expression of lipid genesis genes compared to control (*n* = 9 for 18%; *n* = 8 for 1 and 1%+NAM). **j** Time series of oxygen consumption rate (OCR) measurements by Seahorse XFe96 analyzer. **k** Quantification of OCR (*n* = 4 per group). One-way ANOVA followed by Tukey’s post hoc test. Unless stated differently in the figure legend, all n represents biologically independent samples. Data are shown as the mean ± SEM. Scale bars are as indicated.

To further understand the mechanisms underlying the improved hepatic steatosis in response to NAM treatment, we next evaluated changes in hepatic mitochondrial characteristics in our model. We have previously shown that a protein-deficient diet induces mitochondrial morphological and functional changes and reduces mitochondrial activity in rats under a protein-restricted diet^6^. In our mouse model, immunofluorescent staining of mitochondria in the liver showed that the mitochondria were enlarged but decreased in numbers in the 1% protein-fed mice compared to the 18% protein-fed control group (Fig. 3a). The loss of mitochondria was further confirmed by a significant decrease in the mitochondrial DNA (mtDNA) copy number (Fig. 3b). This feature improved after NAM, NR, and TRP treatment (Fig. 3b and Supplementary Fig. 3a, b). Mitochondrial abundance markers, including TOM20 and HSP60, were both significantly lower in the 1% protein-diet-fed mice compared to the control, but improved with NAM treatment (Fig. 3c, d). The data suggest that NAM treatment can either reduce mitochondria degradation or increase its biogenesis in our model of severe malnutrition.

We also examined hepatic ATP levels and mitochondrial complex proteins, including Complex 1 (NDUFB8), Complex IV (cytochrome c), and Complex V (ATP5A). The livers of the 1% protein-fed malnourished mice had significantly lower hepatic ATP levels compared to the 18% protein-fed control group (Fig. 3e). NAM and other TRP-NAD + pathway modulators significantly restored hepatic ATP levels (Fig. 3e and Supplementary Fig. 3c). Complex I, Complex IV, and Complex V protein levels were significantly lower in the 1% protein-fed group compared to the control group (Fig. 3f, g). Complex IV levels improved significantly after NAM treatment, while no significant change was observed in levels of other complexes. We next assessed the function of isolated liver mitochondria. The results indicated a significantly lower oxygen consumption rate (OCR) after stimulating the respiratory chain to operate at maximum capacity in the untreated 1% protein-fed group compared to the 18% protein-fed control (Fig. 3j, k). For NAM treatment animals, we observed increased maximal respiration levels compared to untreated control of 1% protein-fed animals, suggesting an improvement in mitochondrial fitness (Fig. 3j, k). It should be noted that we did observe in our respirometry experiments similar OCR levels after FCCP (uncoupled state) compared to state 3 (our initial state) after adding ADP, instead of an increase of the maximal respiration (FCCP) levels compared to state 3. This has been observed by others when using isolated mitochondria^23–25^ and could be related to decreasing mitochondrial health over the duration of the assay or the amount of FCCP used as higher levels can lead to inhibition of the respiratory chain. Nevertheless, given the consistent difference in maximal respiration levels between the different conditions, our studies support improved mitochondria activity in NAM-treated animal. Further, we quantified the expression of genes in the β-oxidation and lipogenesis pathway. Expression of the genes in the β-oxidation pathway was reduced in the livers of mice fed a 1% protein diet and was partially restored after NAM treatment, especially *Acaa2* and *Hadha* (Fig. 3h). The expression of lipogenesis genes including *Fasn* and *Acaca* were decreased in mice fed a 1% protein diet (Fig. 3i). NAM supplementation did not influence the mRNA expression of lipogenesis genes (Fig. 3i). Similarly, the abundance of the de novo lipogenesis variables in the lipidomics analyses after rescaling the TG fraction did not change after low-protein feeding and NAM treatment (Supplementary Fig. 5c).

In summary, feeding mice a 1% protein diet altered the hepatic mitochondrial morphology, decreased mitochondrial number and mass, and affected markers of oxidative phosphorylation and β-oxidation. NAM treatment improved the 1% protein-diet-induced mitochondrial changes associated with a recovery in ATP content.

### A low-protein diet leads to changes in hepatic energy metabolism that improve with NAM treatment

**Fig. 4:**
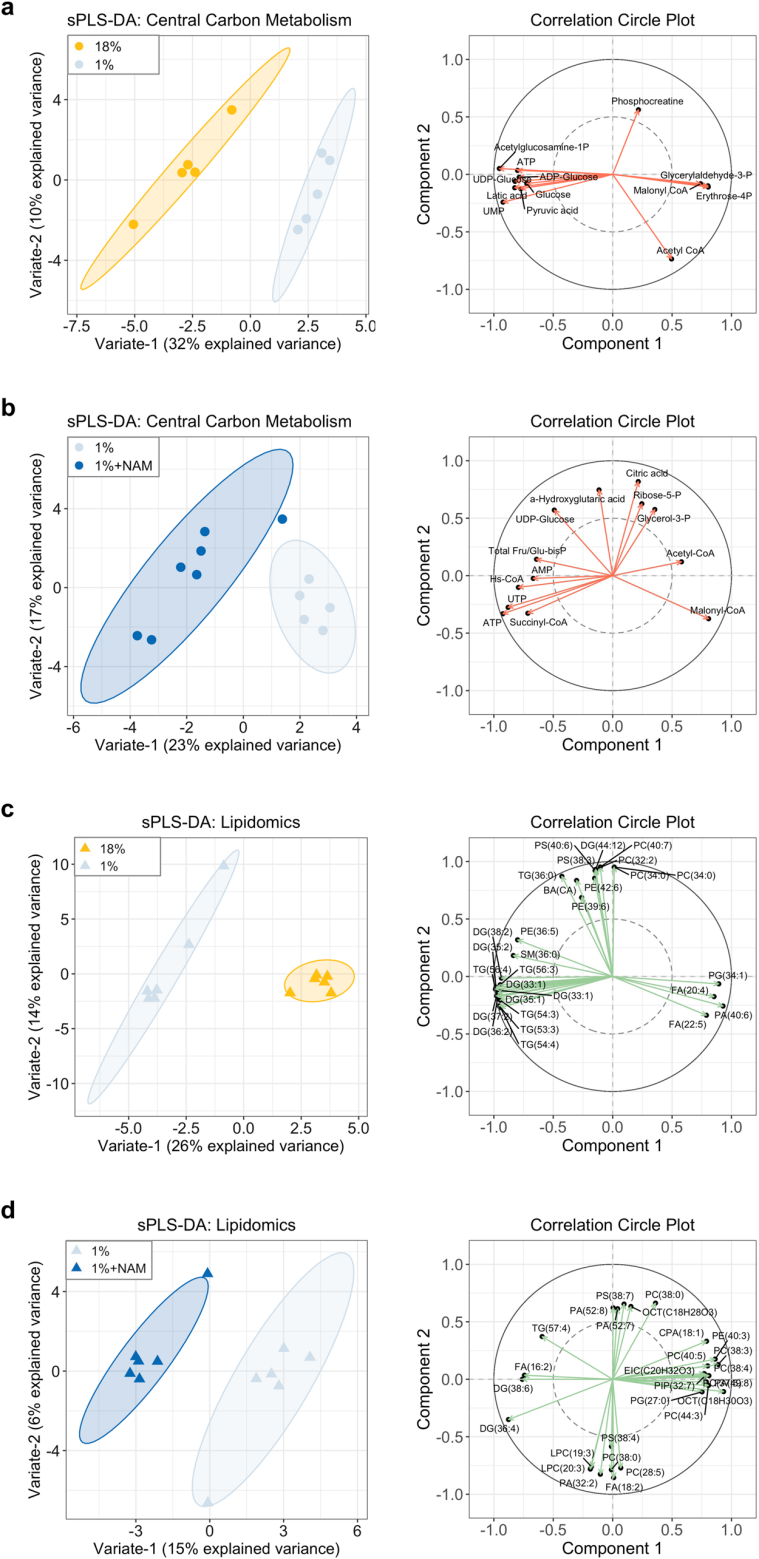
Hepatic metabolomic and lipidomic profiles in mice fed a 18% protein diet or a 1% protein diet with and without NAM treatment. **a** sPLS-DA and correlation circle plots of hepatic central carbon metabolism showing separation of 18 and 1% protein-diet group (*n* = 5 per group). **b** sPLS-DA and correlation circle plots of hepatic central carbon metabolism showing separation of 1% protein diet and NAM-treated group (*n* = 5 for 1%; *n* = 7 for 1%+NAM). **c** sPLS-DA and correlation circle plots of hepatic lipidomics showing separation of 18 and 1% protein-diet group (*n* = 6 per group). **d** sPLS-DA and correlation circle plots of hepatic lipidomics showing separation of 1% protein diet and NAM-treated group (*n* = 6 per group).

To better understand the overall liver metabolic change in mice fed with a 1% protein diet and evaluate the effect of NAM supplementation, we performed a quantitative analysis of liver central carbon metabolism metabolites^26^. The major metabolic profile differences between groups were highlighted by sparse-partial least squares-discriminant analysis (sPLS-DA)^27^. Variable importance in projection (VIP) scores were used to identify the most important metabolites for the clustering. Overall, the hepatic metabolic profiles of the 1% protein-diet-fed malnourished group were clearly separated from those of the 18% protein-diet-fed control group, and distinct from the NAM treatment group (Fig. 4a, b and Supplementary Data 1). Among the metabolomic features, acetylglucosamine-1P, glyceraldehyde-3P, malonyl-CoA, lactic acid, ATP, erythrose-4P, UMP, UDP-glucose, glucose, pyruvic acid, and ADP-glucose mostly discriminated 18% protein diet from 1% protein-diet groups, with variable importance in projection (VIP) score >1 in both components 1 and 2 (Fig. 4a). To be more specific, the 1% protein-fed group showed significantly lower glucose, lactic acid, and pyruvic acid content compared to control (Supplementary Table 1)^28^. GMP and UMP concentrations decreased in the 1% protein-diet-fed group, suggesting disturbed nucleotide metabolism, including pyrimidine and purine synthesis. Malonyl-CoA levels also changed in the 1% protein-diet-fed group, consistent with decreased mitochondrial fatty acid oxidation^29–31^. The overall results were also in line with an earlier report of impaired ATP production and decreased pyruvate uptake, accompanied by altered tricarboxylic acid cycle (TCA) cycle intermediates in a rat model of malnutrition^6^. Modulation of the TRP-NAD + pathway altered hepatic metabolic profiles as observed by sPLS-DA (Fig. 4b and Supplementary Fig. 4a). NAM treatment shifted malonyl-CoA, UTP, ATP, Hs-CoA, UDP-Glucose, total fructose-bisP/glucose-1,6-bisP, acetyl-CoA, AMP, and succinyl-CoA, which mostly differentiate them with 1% protein-diet group (VIP score >1). The concentration of ATP, malonyl-CoA, and acetyl-CoA in the NAM-treated group shifted toward the 18% protein-diet-fed control group, which was related to the improved energy production and carbohydrate and lipid metabolism (Supplementary Table 1)^32^.

To further explore the changes in lipid metabolism in our model and evaluate the effect of TRP-NAD + modulation, we performed lipidomic analyses. Overall, discriminating features were identified that clearly separate the 18% protein diet and 1% protein-diet group, dominated by increased levels of triacylglycerols, diacylglycerols, and sterols (VIP score >1) (Fig. 4c, Supplementary Table 2, and Supplementary Data 1). Interestingly, hepatic phospholipid content was lower in the 1% group compared to the 18% group. The decreased PC/TG ratio and phosphatidylcholines to phosphatidylethanolamines ratio (PC/PE) in the 1% protein-diet group might be linked to the altered energy metabolism and lipid droplet size and dynamics^33,34^. Decreased PC/PE ratios have also been observed in NASH patients^35,36^, potentially through mitochondrial respiratory chain dysfunction and disability to meet energy requirements^37^. NAM treatment clearly separated this group from the 1% protein-diet group, and separation was primarily caused by differences in phosphatidylcholines and diacylglycerols (VIP score >1) (Fig. 4d, Supplementary Table 2, and Supplementary Data 1). NR and TRP treatment groups were close to each other but clearly separate from the MET treatment group, highlighted mainly by altered triacylglycerols and diacylglycerols (with VIP score >1) (Supplementary Fig. 4b and Supplementary Data 1).

### NAM treatment affects NAD + and the SIRT1 pathway in low-protein-fed mice

**Fig. 5:**
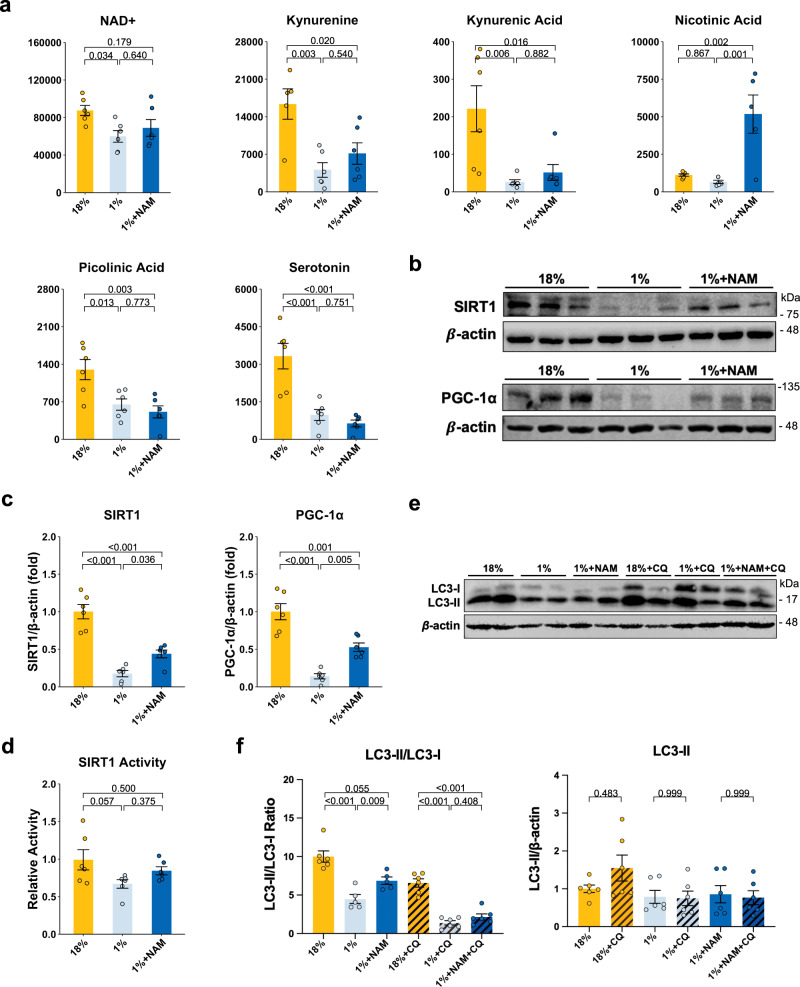
The effect of 1% protein feeding with or without NAM supplementation on TRP-NAD+ pathway metabolites, SIRT1 and downstream targets, and autophagy levels. **a** Hepatic NAD+ levels and TRP-NAD+ pathway metabolites (*n* = 6 per group). **b** Representative SIRT1 and PGC-1α western blots. **c** Quantification of SIRT1 and PGC-1α protein levels in western blots (*n* = 6 per group). **d** SIRT1 activity (*n* = 6 per group). **e** Representative LC3 western blots. **f** Quantification of autophagy marker LC3-II to LC3-I ratio in western blots and LC3-II protein relative to the 18% protein group (*n* = 6 per group) One-way ANOVA followed by Tukey’s post hoc test. Unless stated differently in the figure legend, all *n* represents biologically independent samples. Data are shown as the mean ± SEM.

To determine whether the low-protein diet and NAM treatment directly affect the NAD salvage pathway, we measured the abundance of serum and hepatic NAD+ and tryptophan pathway metabolites of these animals. Serum NAD+ levels in 1% protein-diet feeding animals trended to be lower than the 18% control group (*P* = 0.081) (Supplementary Fig. 5f). It is, however, important to realize that most NAD is intracellular and although changes in serum/plasma NAD levels have been shown to be affected by aging and diet^38,39^, its relevance is not well understood. NAD+ levels and many metabolites in the tryptophan pathway (such as kynurenine, kynurenine acid, and serotonin) in the liver were decreased in the 1% protein-fed mice compared to the 18% protein-fed control group (Fig. 5a). NAM treatment increased hepatic nicotinic acid concentrations, indicating NAM was bioavailable and affected the TRP-NAD+ pathway. However, we did not observe a significant effect of NAM treatment on NAD+ levels itself (*P* = 0.640), whereas NR treatment did significantly increase hepatic NAD+ levels (Supplementary Fig. 3d). This result is consistent with other studies that have reported that NR increased hepatic NAD+ levels^40^. Another chronic NAM supplementation study showed that NAM did not boost NAD+ but enhanced the deacetylation of SIRT1 targets^19^. In addition, we also tested the expression of genes related to this pathway. There was a trend towards decreased *Nnmt*, *Gnmt*, and *Gamt* in the 1% protein-fed animals compared to the control (Supplementary Fig. 5d, e). NAM treatment slightly increased but not significantly impacted *Nnmt* and the methionine cycle (Supplementary Fig. 5d, e).

Next, we investigated changes in the NAD-dependent SIRT1 pathway. The protein levels of SIRT1 and its downstream target PGC-1α were significantly decreased in the mice fed a 1% protein diet compared to the 18% protein-fed control group, and levels of these proteins were significantly improved after NAM treatment, albeit not to the same level as the control group (Fig. 5b, c). SIRT1 activity was lower in the 1% protein-fed group compared to the 18% protein-fed controls, consistent an increased Ac-p53/p53 ratio, whereas the NAM-treated group showed an Ac-p53/p53 ratio similar to the control group (Fig. 5d and Supplementary Fig. 5g, h).

Since SIRT1 has been shown to influence autophagy and we previously showed impairment in autophagy flux in livers of low-protein-fed rodents^6^, we next evaluated autophagy levels by measuring microtubule-associated protein 1A/1B-light chain 3 (LC3) LC3-I and LC3-II protein levels. The autophagy pathway marker of the LC3-II/LC3-I ratio significantly decreased in the 1% protein-fed malnourished group compared to the 18% protein-fed control group, suggesting a decrease in autophagy activation (Fig. 5e, f). However, NAM treatment increased the LC3-II/LC3-I ratio, which suggests an increase in activation of macroautophagy in our model, consistent with activated autophagy after NAM treatment in cell culture systems^14,15^. To address a difference in autophagy flux in the low-protein diet, animals were treated with chloroquine to prevent the lysosomal degradation of autophagosomes. In the control mice, there was a variable response with half of the mice showing an increase in LC3-II levels after 4 h of blocking autophagosome fusion with the lysosome. Low-protein-fed mice did not show increased LC3-II (Fig. 5e, f), suggesting impaired autophagy flux.

Taken together, our results suggest that the TRP-NAD+ pathway is disturbed after feeding a 1% protein diet to mice and that it can be partially restored by NAM treatment. In turn, the improvement in the TRP-NAD+ pathway elevates SIRT1, which may be linked to the increase in PGC-1α and activation of autophagy.

### The effect of NAM on low-protein-diet-induced liver metabolic dysfunction is mediated through SIRT1

**Fig. 6:**
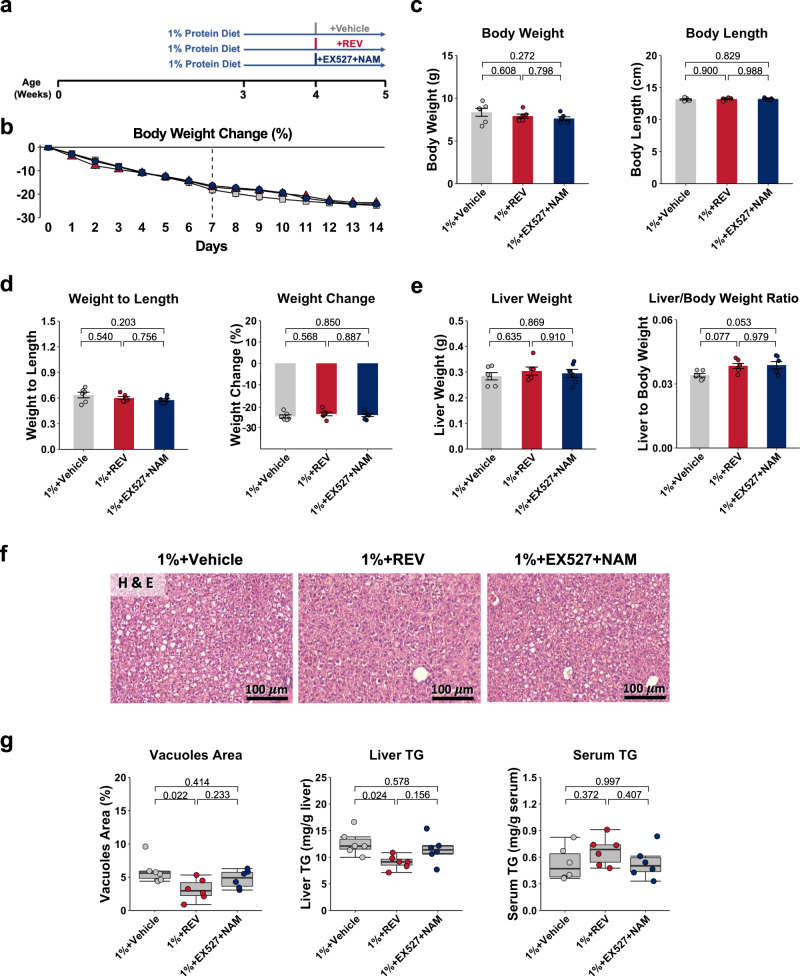
The effect of SIRT1 modulators on basic animal characteristics and hepatic steatosis. **a** Experiment design. **b** Body weight change throughout the experiment (*n* = 6 per group). **c** Average food and liquid intake from day 7 to day 14 (*n* = 6 per group). **d** Final body weight, body length, and weight-to-length ratio assessed at day 14 (*n* = 6 per group). **e** Liver weight, liver weight to body weight ratio (*n* = 6 per group). **f** Representative hematoxylin and eosin staining images of the liver (×20 magnification). The cytoplasm was stained in red, and the nucleus was stained in purple (*n* = 5 replicates). **g** Quantification of vacuoles area and triglyceride (TG) levels (*n* = 6 per group). One-way ANOVA followed by Tukey’s post hoc test. Unless stated differently in the figure legend, all n represents biologically independent samples. Data are shown as the mean ± SEM. Data in box plots are shown as the first to third quartile, whiskers encompass the range, and horizontal lines represent the mean.

To further test whether the effect of NAM is SIRT1-dependent, we performed experiments using SIRT1 modulators in the 1% protein-fed mice with or without NAM supplementation (Fig. 6a). Resveratrol (REV), which indirectly activates SIRT1, was used to investigate if SIRT1 activation was sufficient to demonstrate an improvement in the hepatic metabolic changes caused by 1% protein feeding^41,42^. The SIRT1 inhibitor, selisistat (EX-527)^43,44^, was subsequently used in combination with NAM treatment to determine if the effect of NAM was dependent on the activation of SIRT1.

**Fig. 7:**
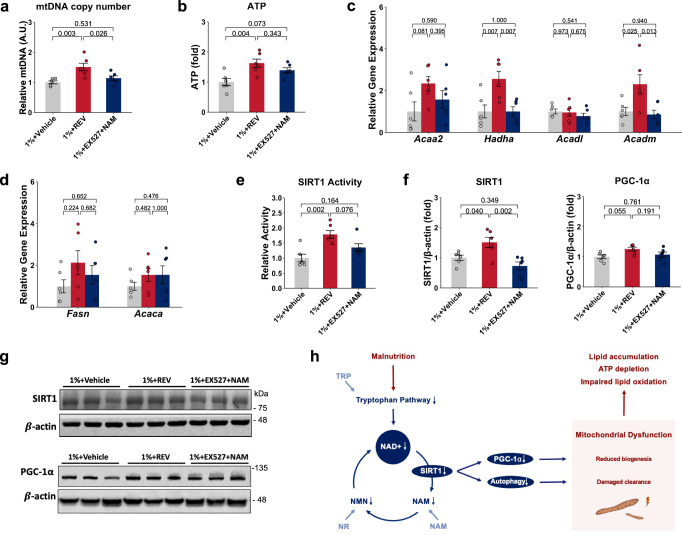
The effect of SIRT1 modulators on hepatic steatosis, mitochondrial characteristics, SIRT1, and its downstream targets. **a** Relative mtDNA copy number (mtDNA/beta-globin) (*n* = 6 per group). **b** ATP levels (*n* = 6 per group). **c** Relative expression of β-oxidation genes (*n* = 6 per group). **d** Relative expression of lipid genesis genes (*n* = 6 per group). **e** SIRT1 activity (*n* = 6 per group). **g** Quantification of SIRT1 and PGC-1α protein levels in western blots (*n* = 6 per group). **f** Representative SIRT1 and PGC-1α western blots. One-way ANOVA followed by Tukey’s post hoc test. Unless stated differently in the figure legend, all *n* represents biologically independent samples. Data are shown as the mean ± SEM. Scale bars are as indicated. **h** Proposed model: In protein malnutrition, decreased tryptophan availability will decrease the tryptophan–kynurenine pathway activity, which is associated with NAD+ and NAM deficiency. This would disturb NAD+ salvage pathway, including SIRT1, influence its downstream target PGC-1α and autophagy, which affect mitochondrial health. These changes lead to ATP depletion and lipid accumulation in the liver. We hypothesize that supplement with TRP-NAD+ modulator would influence NAD+ salvage pathway. This would thereby activate SIRT1, influence PGC-1α and autophagy pathway, which will have a positive effect on mitochondrial biogenesis and clearance of damaged mitochondrial, then improve ATP generation and reduce lipid accumulation in the liver.

Intraperitoneal injection of REV did not change body weight, body length and liver weight compared to the vehicle control group (Fig. 6b–e). However, we observed a decrease in the degree of hepatic steatosis in the 1% protein-fed malnourished group treated with REV, with a nearly twofold decrease in vacuole area and decreased liver TG levels compared to untreated 1% protein-fed animals (Fig. 6f, g). mtDNA copy number and ATP levels significantly increased after REV treatment (Fig. 7a, b). Among the β-oxidation genes, we observed small but significant increases in Hadha and Acadm expression after REV treatment, without a significant change in the expression of lipogenesis genes compared to the vehicle-treated group (Fig. 7c, d). When the 1% protein-fed malnourished mice were treated with both EX-527 and NAM, the effects of NAM treatment on hepatic steatosis and mtDNA copy number were lost (Figs. 6f, g and 7a–d). SIRT1 activity and protein level were upregulated after REV treatment, without significant change in mice treated with EX-527 and NAM (Fig. 7e–g). There was also a trend toward increased PGC-1α protein levels in the REV-treated group (*P* value = 0.055) (Fig. 7f, g). EX-527 with NAM treatment also did not affect SIRT1 and PGC-1α levels compared to the 1% protein-fed malnourished group alone (Fig. 7e). Although REV increased LC3-II/LC3-I ratio and decreased the total 4EBP1, the p-4EBP1/4EBP1 ratio did not show significant change after REV treatment (Supplementary Fig. 6). This result shows that REV activates autophagy and suggests this is independent of mTOR signaling in this low-protein-induced malnutrition model. These data indicate that REV can improve 1% protein-diet-induced hepatic metabolic dysfunction and suggest that the effect of NAM treatment on hepatic metabolism is potentially dependent on the activation of SIRT1.

## Discussion

Our study indicates that feeding weanling mice a 1% protein diet leads to stunted growth, severe wasting, hepatic lipid accumulation, and mitochondrial dysfunction that is associated with a reduction in activity in SIRT1, PGC-1α and autophagy. We demonstrate that supplementing the TRP-NAD+ pathway is able to improve the metabolic phenotype and that this effect is dependent on SIRT1. This study reports the role of the TRP-NAM pathway in the hepatic pathophysiology induced by malnutrition.

The hepatic metabolic changes induced by the protein-deficient diet were consistent with our previous findings in a rat model of severe malnutrition showing liver steatosis and ATP depletion caused by peroxisomal and mitochondrial dysfunction in a rat model of malnutrition^6^. The data are also consistent with limited reports in children with severe malnutrition that have found impaired mitochondrial function^4,5^. Our data indicate that the low-protein-diet-induced steatosis in our malnutrition model is related to mitochondrial dysfunction. Hepatic mitochondrial morphological and functional changes have been reported previously in severely malnourished children^5,45^. Previous clinical studies have suggested that hepatic steatosis in malnourished children is not associated with increased lipogenesis, potentially related to well-documented impaired insulin secretion in this population^46,47^. The gene expression and lipidomic data in our model are consistent with data observed in malnourished children that lipogenesis is not a factor in the development of hepatic steatosis. Furthermore, hepatic lipid secretion has also been reported to be not affected in children with severe malnutrition. The steatosis might, in part, be explained by enhanced lipolysis, also given the reduced insulin secretion^48,49^. Limited historical data exists describing the effects of insufficient niacin on pellagra patients, which is marked by fat and iron deposition on the liver^50,51^. Interestingly, debate on whether the phenotypical features of severe malnutrition resembled those of pellagra were discussed in the early publications on severe malnutrition^52,53^. Besides, there is considerable overlap with features seen in patients with NAFLD, including changes in mitochondrial complexes, mitochondrial biogenesis, and hepatic lipid accumulation^54–56^. The reduction in mitochondrial mass seen in our mouse model is different from previous observations in low-protein-fed rats, where an increase in mitochondrial mass was observed^6^. However, the reduction in mtDNA in our low-protein-diet mouse model was consistent with another previous report in fetal and early postnatal malnourished rats fed a low casein diet^57^.

The reduction in mitochondrial mass and mtDNA in low-protein-fed mice was associated with a reduction in PGC-1α, a well-known regulator of cellular energy metabolism and activator of mitochondrial biogenesis^58,59^. PGC-1α can co-activate transcription factors such as peroxisome proliferator-activated receptor (PPARα) and nuclear respiratory factors (NRF1 and NRF2) to regulate mitochondrial biogenesis and fatty acid oxidation^60^. Mice that are deficient in PGC-1α have impaired energy metabolism that is related to a decrease in mitochondrial number and respiratory capacity^61^. This suggests that the reduction in mitochondrial mass is related to a decrease in mitochondrial biogenesis upon low-protein feeding. The changes in mitochondrial morphology, mitochondrial complex content, and markers of mitochondrial function, such as ATP, also indicate that the mitochondria that are present in the liver after a period of low-protein feeding are damaged and dysfunctional. Mitochondrial degradation is regulated through a selective autophagy process called mitophagy^13^, and our data suggest that autophagy activation is decreased during nutritional stress. This could contribute to a high relative content of damaged mitochondria that would normally have been degraded through mitophagy. NAM treatment increased PGC-1α protein levels, mitochondrial mass and content of mitochondrial complexes while activating the autophagy pathway, suggesting a rebalancing of mitochondrial biogenesis and mitophagy.

PGC-1α and autophagy are both regulated by SIRT1. SIRT1 directly deacetylates PGC-1α at multiple lysine sites, and the induction pattern of SIRT1 protein correlates with the expression of PGC-1α^62^. In addition, SIRT1 regulates autophagy by acting on multiple autophagy effectors. These mechanisms include directly inducing autophagy by deacetylating autophagy-related genes (ATGs) and LC3, indirectly inhibiting the mTOR pathway by activation of AMP-activated protein kinase (AMPK), as well as modulating the expression of autophagy and mitophagy regulatory molecules (e.g. Rab7 and Bnip3) through deacetylation of Forkhead box O transcription factors (FOXOs)^63,64^. SIRT1 levels were decreased in our low-protein-diet-fed mice. As SIRT1 activity is dependent on NAD availability, we propose that lower SIRT1 activity is associated with reduced levels of NAD and other metabolites in the TRP-NAD+ pathway in low-protein-diet-fed mice. Supplementing protein-deficient animals with NAM was found to rescue SIRT1-mediated pathways. We propose that the reduction in NAD prevents the SIRT1-mediated activation of PGC-1α and autophagy pathway. Our results are consistent with a clinical study reporting that increased malnutrition risk was associated with decreased SIRT1 expression^65^. The decreased protein levels of SIRT1 found after low-protein feeding could potentially be explained by diet-triggered cleavage of SIRT1 protein. For example, a high-fat diet has been shown to induce SIRT1 protein cleavage leading to metabolic dysfunction^66^.

NAM was shown to increase SIRT1 levels. The effect was not specific to NAM, as NR and TRP demonstrated a similar effect. Other NAD+ precursors such as NR and TRP have demonstrated a similar effect in the previous studies^18,67,68^. We focused on NAM specifically for more in-depth investigations because of its low cost and excellent safety profile. Treatments with NAM and other NAD+ precursors have shown beneficial effects in various metabolic dysfunction models, including fatty liver, obesity, metabolic syndrome, and diabetes^19,69,70^. The beneficial effects in these studies have been related to an improved mitochondrial function, mediated by NAD+-dependent sirtuin activation^18,67,68^. Our SIRT1 modulation experiments demonstrated that in our malnutrition model, the effects of NAM were dependent on the presence of SIRT1 and that stimulating SIRT1 was sufficient to produce beneficial effects on mitochondrial function. The results are consistent with studies in high-fat-fed mice where resveratrol impacted mitochondrial function and prevented hepatic steatosis^41^.

In our study, NAM treatment did not significantly restore NAD+ levels whereas NR did, however NAM improved SIRT1 and PGC-1α levels. Some studies have shown that NAM has the ability to increase cellular and blood NAD+ content in different metabolic disorder models (e.g. NAFLD mice, hepatocytes with endoplasmic reticulum stress)^71–74^. However, other studies have found no direct effect of NAM supplementation on NAD+ levels^19,75^. If the extra NAD that is synthesized is readily used for deacetylation, then you would not see a significant increase. These differences in findings might also be related to the duration and variation in the dose of NAM and the animal models used affecting NAM metabolism. For example, NAM can affect SIRT1 activity differently by acting as an NAD+ precursor and a non-competitive end-product inhibitor^76^. In addition, NAM clearance pathways through MNAM-mediated SIRT1 protein stabilization can also regulate hepatic nutrient metabolism^77,78^. The dose of NAM used was in a similar range as used previously in mice, but higher than used when compared to limited human trials^70,79,80^.

Although, we provide evidence for a role of SIRT1 in the altered mitochondrial homeostasis and function in malnutrition, we cannot exclude SIRT1-independent effects. Resveratrol has been reported to affect a number of enzymes, such as AMPK and the AMPK substrate acetyl-CoA carboxylase (ACC) and possesses intrinsic antioxidant capacity^81,82^. Although EX-527 has been shown to be a selective SIRT1 inhibitor, but there might be off-target effects^83^. Future studies could make use of liver-specific SIRT1 knock-out models to validate our findings. Both NAM and REV treatment did not recover body weight and body length in our malnourished diet model, indicating a lack of effect on growth, consistent with early studies^84–86^. Besides, malnutrition is associated with profound changes in the microbiome and could also impact metabolic function in malnutrition^87^. We also cannot exclude NAM treatment affected hepatic metabolism, in part, by altering the intestinal microbiome.

In conclusion, this work provides evidence for the role of the TRP-NAD+ pathway in liver metabolic dysfunction in a mouse model of severe malnutrition, potentially mediated through changes in levels of SIRT1. This study improves our understanding of the cellular pathophysiology of severe malnutrition, and future investigation is needed. The results of this project could lead to the development of new interventions that target the TRP-NAD+ pathway, which could then be taken to clinical trials.

## Methods

### Animals and diets

A breeding colony of C57BL/6 mice was obtained from Jackson Laboratories (Bar Harbor, ME, USA). Male mice at 3 weeks postpartum were weaned and housed socially in filtered cages at The Hospital for Sick Children (Toronto, ON, Canada). Weanling male C57BL/6J mice were randomized into different groups fed with control diet (18% protein, ENVIGO, USA) or malnourished diet (1% protein, ENVIGO, USA) for a period of 2 weeks (Supplementary Fig. 7 and Supplementary Table 5). The decrease in casein protein was compensated with an increase in corn starch (Supplementary Table 5). After 7 days, malnourished subgroups were treated with modulators of the TRP-NAD+ pathway until sacrifice on day 14. NAM, NR, and TRP (Sigma-Aldrich, USA) were given by drinking water in a dose of 160 mg/kg body weight/day, and methionine (Sigma-Aldrich, USA) was included in diets at a concentration of 0.75 g/kg diet^16,75,80^. In a subset of mice, after 1% protein diet for 7 days, intraperitoneal injections treated with either resveratrol (25 mg/kg/d; Sigma-Aldrich, USA) or EX-527 (10 mg/kg/d; Sigma-Aldrich, USA) with nicotinamide were given for 7 consecutive days until sacrifice^43,44,88^. For the chloroquine treatment experiment, animals were administered with chloroquine (50 mg/kg; Abcam, USA) by intraperitoneal injection 4 h before sacrifice on day 14. All groups were housed in a temperature-controlled environment (23 °C), humidity (40–60%), light-dark cycle (6 am–8 pm light) and had ad libitum access to diet and water throughout the study. Mice were checked every other day for body weight, dehydration, and lethargy to ensure humane endpoints were not reached. After treatment, mice were anesthetized with isoflurane anesthesia and were sacrificed through subsequent exsanguination. All animal experiments were approved by the Animal Care Committee of The Hospital for Sick Children, Toronto (Animal Use Protocol Number: 1000030900).

### Body parameters

Body weight, food intake, and liquid intake were monitored from day 1 to day 14. At the end of the experiment (day 14 post weaning), mice were fasted from 7:00 am for 4 h and then humanely euthanized, after which liver and blood were rapidly collected. Final body weight, body length, and liver weight were recorded. Blood was collected by cardiac puncture. Liver tissue was collected for histology or stored at −80 °C for later use in biochemical analyses. Glucose concentration was determined via tail snip at 0 h, 4 h, 8 h, and 12 h fasting in the daylight cycle, using an automatic freestyle glucometer (Abbott, USA). Serum levels of albumin were measured using a mouse albumin ELISA kit (ab207620, Abcam, USA) following the standard manufacturer’s protocol. Metabolic rate was assessed by indirect calorimetry using the Columbus Instruments (Oxymax Lab Animal Monitoring System: CLAMS, USA)^19^.

### Histology

Fresh livers tissues were fixed in 4% paraformaldehyde (PFA) overnight at 4 °C and then embedded in either paraffin or optimum cutting temperature (OCT) compound. Liver paraffin sections (5 µm) were stained with hematoxylin and eosin (H&E) for morphology. Quantification of vacuoles (white area) in H&E stained sections was conducted using ImageJ 1.52 v and Python v3.7.2. To confirm results, liver cryosections were stained with Oil red O (10 µm) for lipid droplets. Slides were visualized under a light microscope and were measured using Panoramic Viewer version 1.15 software (3DHISTECH Ltd, Hungary). For each slide, at least five pictures were captured.

### Immunofluorescence

Liver cryosections with 4-µm thickness were stained with 1:1000 fluorinated boron-dipyrromethene (BODIPY) to visualize fat droplets. An HSP60 antibody (1:1000) was used to visualize mitochondrial morphology. Nuclei were counterstained with DAPI (1:1000). Slides were mounted with a mounting medium (Vector Laboratories Inc., Burlington, Canada), and images were acquired on a Nikon Spinning Disk Confocal Microscope (Nikon Inc., USA). Additional information can be found in Supplementary Table 3.

### Plasma tryptophan analysis

Plasma samples were mixed with equal volumes of norleucine as the internal standard. Samples were centrifuged at 20,238×*g* for 5 min and subsequently measured on Biochrom 30+ Amino Acid Analyzer (Biochrom, UK).

### Serum NAD+ analysis

Freshly collected serum samples were used for NAD+ analysis. Serum NAD+ concentrations were determined by a commercially available NAD/NADH Assay Kit (ab65348, Abcam, USA) following the standard manufacturer’s protocol.

### Seahorse analysis

Seahorse experiments were performed on freshly isolated liver mitochondria from mice under different treatments. Mice were fasted for 4 h and liver mitochondria were isolated by differential centrifugation as described previously^89^. Briefly, mice liver was dissected in an ice-cold mitochondrial isolation buffer (100 mM Tris-MOPS, 10 mM EGTA/Tris and 90 mM sucrose, pH 7.4). Then, the liver was minced into small pieces, washed out of the blood, and homogenized by a glass Dounce tissue homogenizer. The pellet containing mitochondrial was isolated by centrifugation at 7000×*g* and 4 °C and resuspended in the isolation buffer. The mitochondrial concentration was determined using a Pierce BCA Protein Assay Kit (Thermo Fisher Scientific, USA). Real-time measurements of oxygen consumption rates (OCR) were conducted using the Seahorse XFe96 analyzer (Agilent, USA) according to the manufacturer’s protocol^90,91^. Briefly, 3 µg liver mitochondria per well with six replicates per sample were plated on 96-well Seahorse mini plates. After cartridge calibration, the plate was loaded and assayed for OCR (pmol O_2_/min) for respiration at the basal level and after sequential injection of oligomycin (2.5 μg/mL), carbonyl cyanide-p-trifluoromethoxyphenylhydrazone (FCCP) (2.2 μM), and antimycin A (2 μM), initiated by 1 mM ADP and with 10 mM succinate and 2 μM rotenone (as the substrate) per well, according to the manufacturer’s protocol. Three continuous measurements were conducted after each injection. Results were obtained and calculated by Wave 2.6.1.53 software (Agilent, USA), according to the manufacturer’s instructions.

### Western blotting

Western blot analysis was conducted to measure the protein levels. Liver tissue protein was extracted through sonication of tissue with extraction buffer and protease inhibitor cocktail (Sigma-Aldrich, USA). The protein concentration was measured using a Pierce BCA Protein Assay Kit (Thermo Fisher Scientific, USA). In total, 20 µg of samples were electrophoresed through gels made in-house by layering 3 polyacrylamide concentrations (4%, 8 and 12%) and transferred onto a polyvinylidene fluoride (PVDF) membrane. Membranes were probed with 1:1000 dilutions of anti HSP60 (Abcam, USA), TOM20 (Santa Cruz, USA), Complex I (Abcam, USA), Complex IV (Santa Cruz, USA), Complex V (Abcam, USA), 4EBP1 (Cell Signaling, USA), p-4EBP1 (Cell Signaling, USA), and LC3B (Cell Signalling, USA). 1:500 dilutions of SIRT1 (Cell Signalling, USA), PGC-1α (Abcam, USA), Ac-p53 (Cell Signalling, USA), and p53(Cell Signalling, USA). β-actin (Abcam, USA) was used as a loading control in 1:1000 dilution. Then proteins were visualized using a Pierce enhanced chemiluminescence (ECL) Plus Kit (Invitrogen, USA). Western blot quantification was performed using Image Studio (LI-COR Biosciences, USA). After quantification, Restore™ Western Blot Stripping Buffer (Thermo Fisher Scientific, USA) was used to remove primary and secondary antibodies, allowing the same membrane to be re-used for a different antibody. Uncropped raw blots are provided in Source Data. Additional information can be found in Supplementary Table 3.

### qPCR

Total RNA was isolated from frozen liver tissue using a Direct-zol RNA Miniprep Kit (ZYMO Research Inc., USA). cDNA was synthesized by a Super Script VILO cDNA Synthesis Kit (Thermo Fisher Scientific, USA). In total, 500 ng of liver total RNA were used for cDNA synthesis. Ribosomal protein l13a (Rpl13a) was used as the reference gene. qPCR was performed on CFX384 Touch Real-Time PCR Detection System (Bio-Rad, USA). For mtDNA copy number measurements, 500 ng of genomic DNA were used for each qPCR reaction and β-globin was used as reference^92^. Additional information can be found in Supplementary Table 4.

### SIRT1 activity assay

SIRT1 activity was quantified with a SIRT1 Activity Fluorometric Assay Kit (Abcam, USA) following the manufacturer’s instructions^93^. Protein concentrations were determined using a Pierce BCA Protein Assay Kit (Thermo Fisher Scientific, USA). In duplicates, 60 µg samples were added to the black 96-well plates per assay reagent. Fluorescence intensity (excitation 350 nm, emission 460 nm) was measured by Varioskan Lux Multimode Microplate Reader (Thermo Fisher Scientific, USA). Reactions were stopped after 15 min, and results are reported as fold change in relative fluorescence units (RFU) compared to controls.

### Metabolomic analysis

Targeted metabolomic profiling (pathway-specific assays) was performed by The Metabolomics Innovation Centre (TMIC, Canada). The quantitation of central carbon metabolism metabolites in mouse liver was measured by ultraperformance liquid chromatography-tandem mass spectrometry (UPLC-MS/MS). A Dionex 3400 UHPLC system coupled to a 4000 QTRAP mass spectrometer, was implemented for the analysis. The MS instrument was used in the multiple reaction monitoring (MRM) mode with negative-ion (−) or positive-ion (+) detection, depending on which group of metabolites was measured. Each liver tissue sample was frozen and placed into an Eppendorf tube. Water, at 2 μL per mg tissue, was added and the samples were homogenized for 1 min twice at a shaking frequency of 30 Hz, with the aid of two 4-mm metal balls, on a MM 400 mill mixer. After a short-time centrifuge, methanol, at 8 μL per mg tissue, was added and the samples were homogenized again for 1 min twice using the same settings. The samples were then sonicated in an ice-water bath for 3 min, followed by centrifugal clarification at 20,238×*g* and 5 °C in an Eppendorf 5424R centrifuge for 20 min. The clear supernatants were collected to conduct quantitation of TCA cycle carboxylic acids, glucose and selected sugar phosphates, and other phosphate-containing metabolites and nucleotides by UPLC-MS/MS. Concentrations of the detected metabolites were calculated from their linear-regression calibration curves with internal calibration. Tryptophan pathway metabolites were also measured using a UPLC-MS-based targeted method and arbitrary units were reported^94^.

### Lipidomic analysis

Lipidomic analysis was performed at Core Metabolomics and Lipidomics Laboratory, Wellcome Trust-Metabolic Research Laboratories (University of Cambridge, UK). Liver samples were homogenized, lipids were extracted according to a published procedure, and data was acquired through Direct Infusion Mass Spectrometry (DI-MS)^95^. Briefly, liver sections (30 mg/each) were homogenized (Tissue homogeniser II, Qiagen) in a buffer of chaeotropes (guanidinium chloride (6 M) and thiourea (1.5 M) in deionised water, 500 µL/sample). The liver homogenates (30 μL) were injected into a well (96-well plate, Esslab Plate+ ™, 2.4 mL/well, glass-coated) followed by methanol spiked with internal standards (150 µL), water (500 µL), and DMT (500 µL, dichloromethane, methanol and triethylammonium chloride, 3:1:0.005). Most of the aqueous solution was removed (96-channel pipette). A portion of the organic solution (20 µL) was transferred to a high throughput plate (384 w, glass-coated, Esslab Plate+ ™) before being dried (N_2_ (g)). The dried films were re-dissolved (TBME, 30 µL/well) and diluted with a stock mixture of alcohols and ammonium acetate (100 µL/well; propan-2-ol: methanol, 2:1; CH_3_COONH_4_ 7.5 mM). The analytical plate was heat-sealed and run immediately. Lipid fraction isolates were profiled using a three-part method in DI-MS^95^. All samples were infused into an Exactive Orbitrap (Thermo, Hemel Hampstead, UK), using a TriVersa NanoMate (Advion, Ithaca, USA). Samples (15 μL) were sprayed at 1.2 kV in the positive-ion mode. The Exactive started acquiring data 20 s after sample aspiration began. The Exactive acquired data with a scan rate of 1 Hz (resulting in a mass resolution of 100,000 full width at half-maximum [FWHM] at 400 *m/z*). The Automatic Gain Control was set to 3,000,000, and the maximum ion injection time to 50 ms. After 72 s of acquisition in positive mode the NanoMate and the Exactive switched over to negative ionization mode, decreasing the voltage to −1.5 kV and the maximum ion injection time to 50 ms. The spray was maintained for another 66 s, after which the NanoMate and Exactive switched over to negative mode with in-source fragmentation (also known as collision-induced dissociation, CID; 70 eV) for a further 66 s. After this time, the spray was stopped and the tip discarded, before the analysis of the next sample began. The sample plate was kept at 15 °C throughout the acquisition. Samples were run in row order. The instrument was operated in full scan mode from *m/z* 150–1200 Da (for singly charged species).

### Statistical analysis

Statistical significance for the difference between two groups was calculated by using an unpaired two-tailed Student’s *T* test. Statistical significance for the difference among more than two groups was calculated by using an ordinary one-way ANOVA followed by the Turkey’s post hoc test^96^. Statistical analysis was performed with R software (version 3.5.2), Prism GraphPad (version 9.0), and MetaboAnalyst (version 4.0). Statistical significance was given as *P < 0.05, **P < 0.01, and ***P < 0.001. To account for multiple testing, *P* values were adjusted using a false discovery rate (FDR) correction with a 0.05 cutoff in omics analyses. The results are expressed as mean ± standard error of the mean (SEM) unless otherwise indicated.

### Reporting summary

Further information on research design is available in the Nature Portfolio Reporting Summary linked to this article.

## Supplementary information

Supplementary Information

Description of Additional Supplementary Files

Supplementary Data 1

Reporting Summary

## Data Availability

All relevant data of this study are available within the paper and its supplementary information files, and are available from the corresponding author upon request without restrictions.  Source data are provided with this paper.

## Source data

Source Data
