# Supplementary material for: The role of the tryptophan-NAD + pathway in a mouse model of severe malnutrition induced liver dysfunction
Source: Nat Commun. 2022 Dec 8;13:7576. doi: 10.1038/s41467-022-35317-y (PMC9732354; doi:10.1038/s41467-022-35317-y)
Supplement: Supplementary file 1 — Supplementary Information [file 41467_2022_35317_MOESM1_ESM.pdf]

SUPPLEMENTARY INFORMATION

The role of the tryptophan-NAD+ pathway in a mouse model of severe malnutrition induced liver dysfunction

Guanlan Hu[1,2], Catriona Ling[1,2], Lijun Chi[2], Mehakpreet K. Thind[1,2], Samuel Furse[3,4], Albert Koulman[3], Jonathan R. Swann[5,6], Dorothy Lee[2], Marjolein M. Calon[2], Celine Bourdon[2,7], Christian J. Versloot[8], Barbara M. Bakker[8], Gerard Bryan Gonzales[2,9], Peter K. Kim[10,11] & Robert H. J. Bandsma[1,2,7,8,12] *

SUPPLEMENTARY FILES:

Supplementary Figure 1

Supplementary Figure 2

Supplementary Figure 3

Supplementary Figure 4

Supplementary Figure 5

Supplementary Figure 6

Supplementary Figure 7

Supplementary Table 1

Supplementary Table 2

Supplementary Table 3

Supplementary Table 4

Supplementary Table 5

Supplementary Figure 1

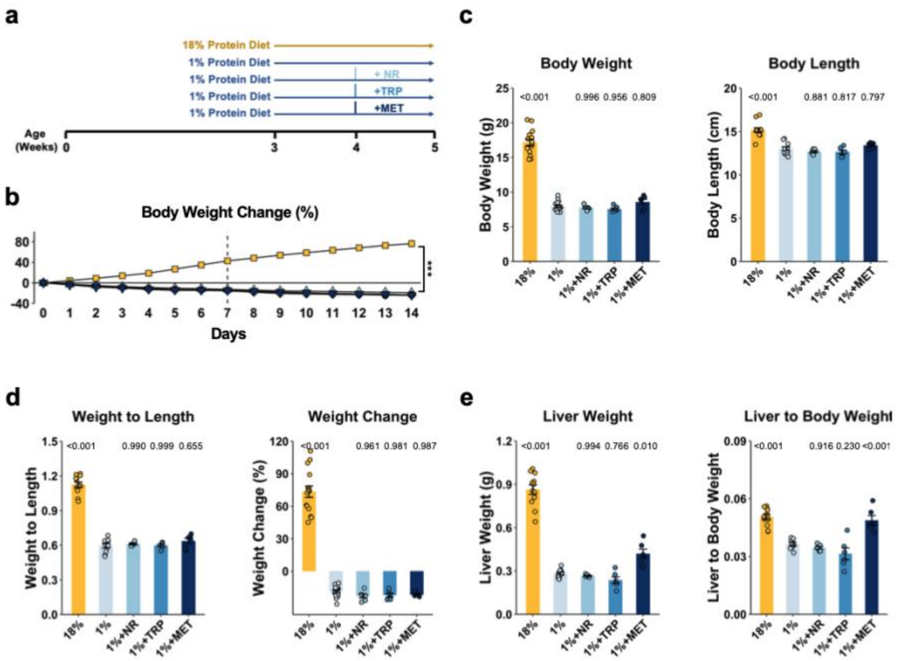

Supplementary Fig. 1 Feeding a 1% protein diet with or without NR, TRP, and MET supplementation on basic animal characteristics. **a** Experimental design of NR, TRP and MET supplementation. **b** Body weight change throughout the experiment (n=15 for 18% and 1%; n=6 for all other groups). **c** Final body weight and body length assessed at day 14 (n=12 for 18%; n=10 for 1%; n=6 for all other groups). **d** Weight to length ratio and weight change assessed at day 14 (n=12 for 18%; n=10 for 1%; n=6 for all other groups). **e** Liver weight, liver weight to body weight ratio (n=12 for 18%; n=10 for 1%; n=6 for all other groups). One-way ANOVA followed by Tukey's post hoc test was used to compare the different groups. Unless stated differently in the figure legend, all n represents biologically independent samples. Data are shown as the mean ± S.E.M.

Supplementary Figure 2

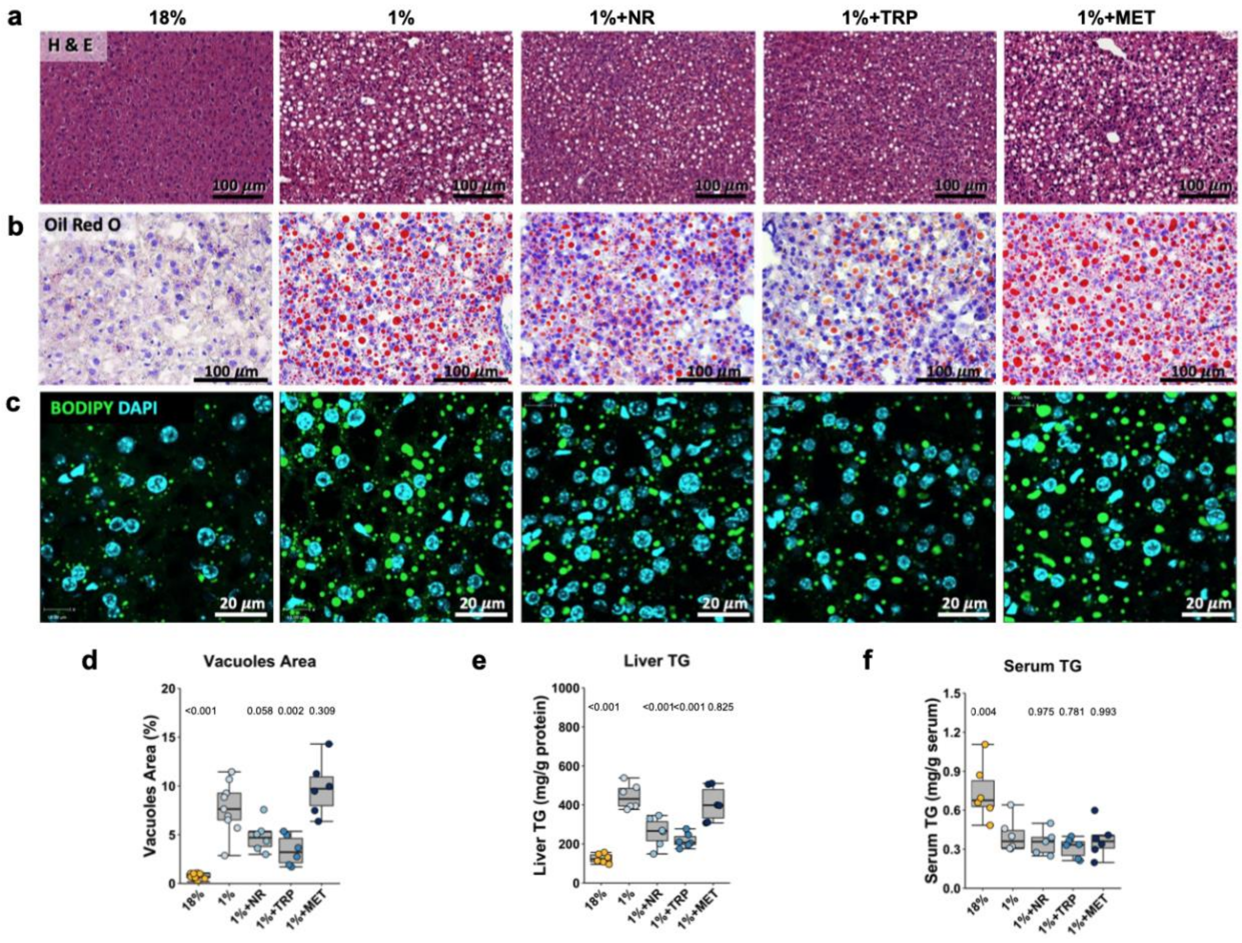

Supplementary Fig. 2 Liver histology in mice fed an 18% or 1% protein diet after NR, TRP and Met supplementation. **a** Representative hematoxylin and eosin staining images of the liver (20X magnification). Cytoplasm was stained in red, and nucleus was stained in purple (n=3 biological replicates per group). **b** Representative oil red o stain staining images of the liver (20X magnification). Fat droplet was stained in red, and nucleus was stained in purple (n=3 biological replicates per group). **c** Representative immunofluorescence images of the liver (40X magnification). BODIPY was used to stain fat droplet in green, and DAPI was used to counter stain nucleus in blue (n=3 biological replicates per group). **d** Quantification of vacuoles area (n=9 for 18% and 1%; n=6 for all other groups). **e** Liver triglyceride (TG) concentrations (n=6 per group). **f** Serum TG concentrations (n=6 per group). One-way ANOVA followed by Tukey's post hoc test was used to compare the different groups. Unless stated differently in the figure legend, all n represents biologically independent samples. Data in box plots are shown as the first to third quartile, whiskers encompass the range, and horizontal lines represent the mean. Scale bars are as indicated.

Supplementary Figure 3

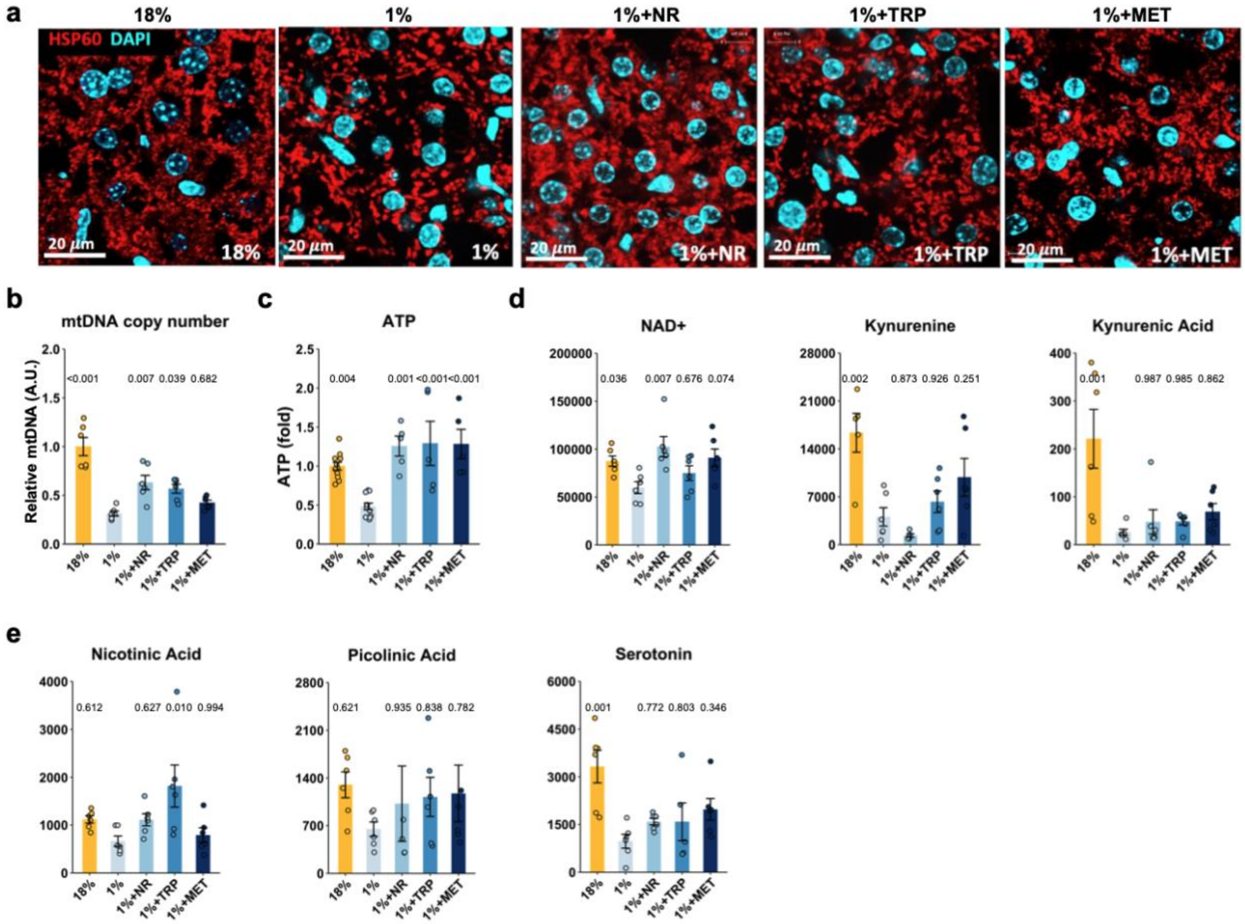

Supplementary Fig. 3 Mitochondrial characteristics and TRP-NAD+ pathway metabolites in mice fed an 18% protein or 1% protein diet and supplemented with NR, TRP or MET. **a** Representative immunofluorescence images of mitochondrial (60X magnification). HSP60 was used to stain mitochondrial in red, and DAPI was used to counter stain nucleus in blue (n=3 biological replicates per group). **b** Relative mtDNA copy number (mtDNA/beta-globin) (n=6 per group). **c** ATP levels (n=11 for 18% and 1%; n=5 for 1%+NR, 1%+TRP, and 1%+MET). **d, e** Hepatic NAD+ levels and TRP-NAD+ pathway metabolites (n=6 per group). One-way ANOVA followed by Tukey's post hoc test was used to compare the different groups. Unless stated differently in the figure legend, all n represents biologically independent samples. Data are shown as the mean ± S.E.M. Scale bars are as indicated.

Supplementary Figure 4

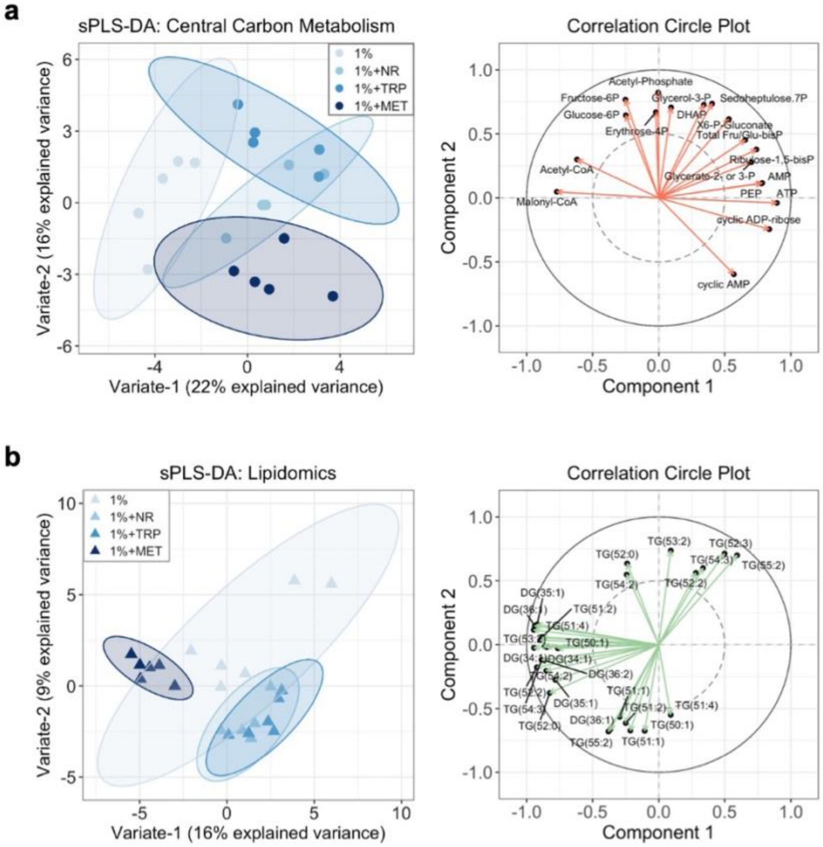

Supplementary Fig. 4 Hepatic metabolic and lipidomic profiles after treatment with TRP-NAD+ pathway modulators. **a** sPLS-DA and correlation circle plots of hepatic central carbon metabolism showing separation of different treatments (n=5 per group). **b** sPLS-DA and correlation circle plots of hepatic lipidomics showing separation of different treatments (n=6 per group).

Supplementary Figure 5

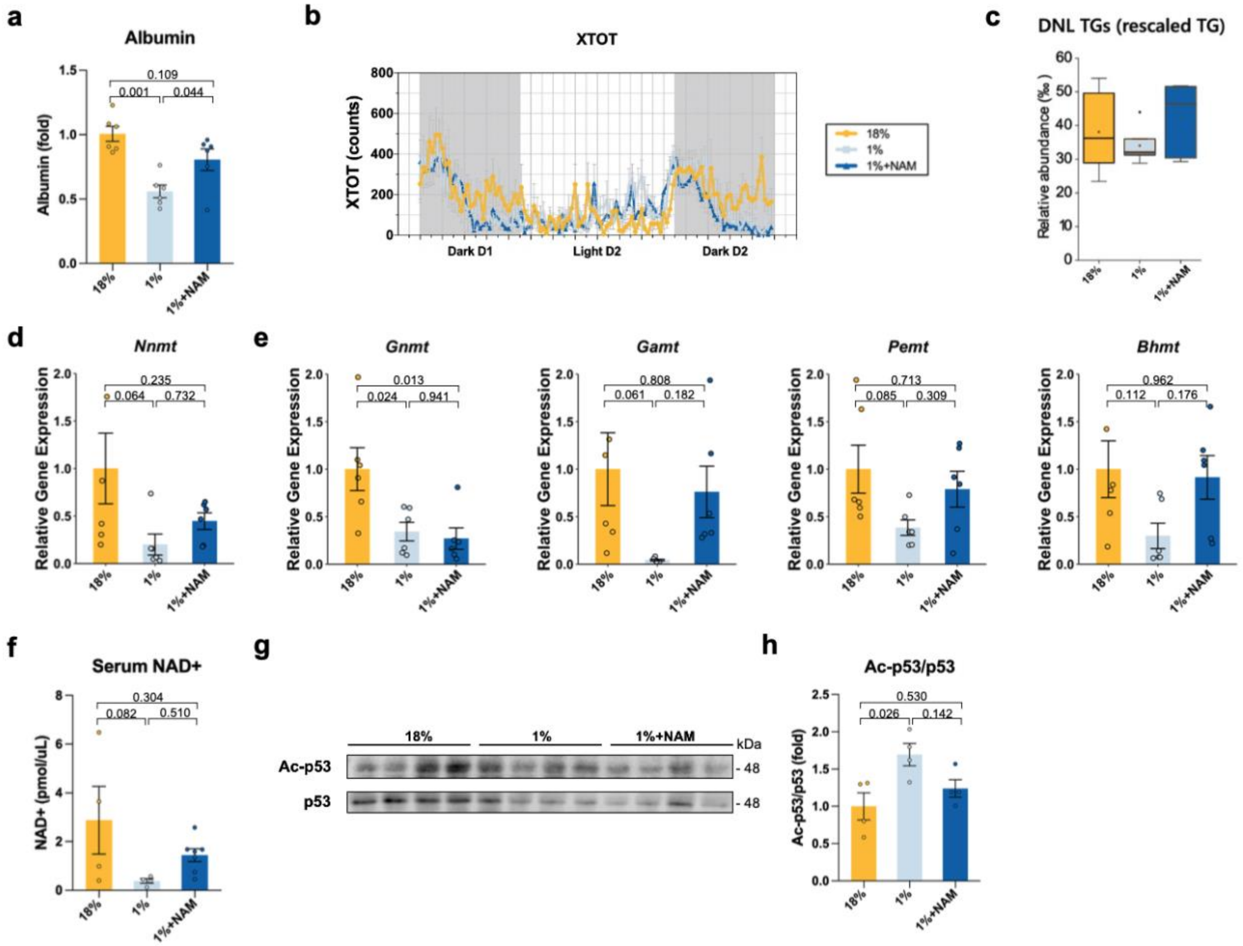

Supplementary Fig. 5 a Serum albumin levels (n=6). **b** Total movement (XTOT, counts of laser breaks) (n=7 for 18%; n=6 for 1%; n=7 for 1%+NAM). **c** Abundance of the de novo lipogenesis (DNL) variables in lipodomics after rescaling the TG fraction (n=6 per group). **d** Relative expression of *Nnmt* gene compared to control (n=6 per group). **e** Relative expression of *Gnmt*, *Gamt*, *Pemt*, and *Bhmt* genes compared to control (n=6 per group). **f** Serum NAD+ levels (n=4 for 18%, n=4 for 1%, n=7 for 1%+NAM). **g**, **h** Western blots and quantification of Ac-p53/p53 (n=4 per group). One-way ANOVA followed by Tukey's post hoc test was used to compare differences between the groups. Unless stated differently in the figure legend, all n represents biologically independent samples. Data are shown as the mean ± S.E.M. Scale bars are as indicated.

Supplementary Figure 6

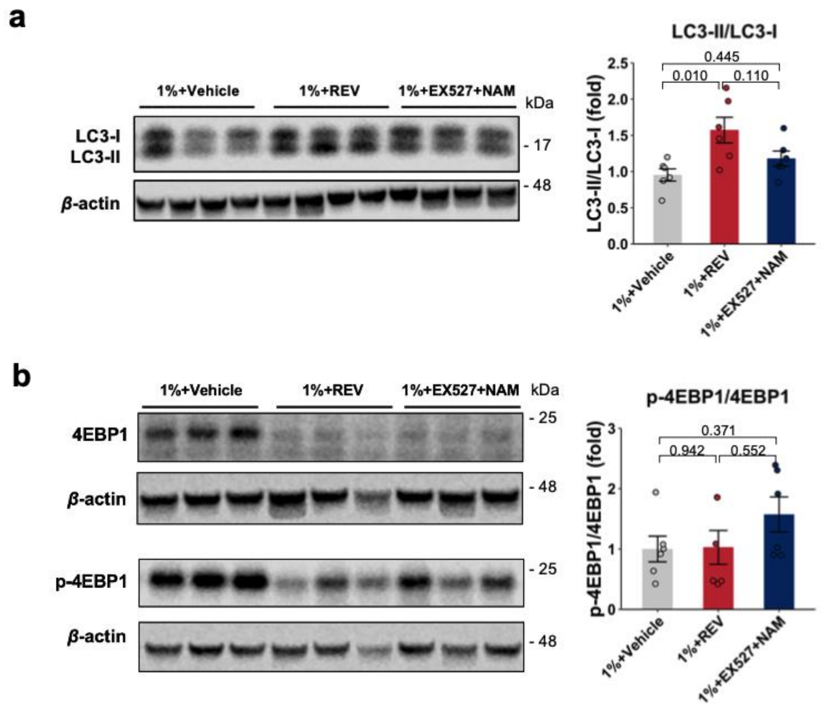

Supplementary Fig. 6 a Representative LC3 western blots and quantification of LC3-II to LC3-I ratio (n=6 per group). **b** Representative 4EBP and p-4EBP western blots and quantification of p4EBP/4EBP (n=6 per group). One-way ANOVA followed by Tukey's post hoc test was used to compare differences between the groups. Unless stated differently in the figure legend, all n represents biologically independent samples. Data are shown as the mean ± S.E.M.

Supplementary Figure 7

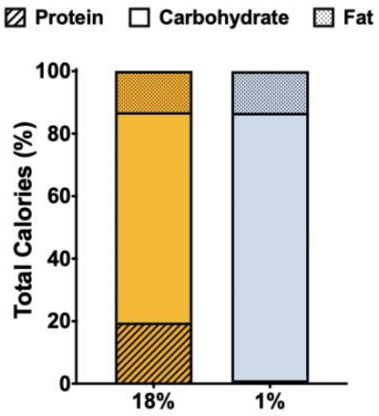

Supplementary Fig. 7 Total diet calories.

Supplementary Table 1

Supplementary Table 1 Hepatic central carbon metabolism profiles under different treatments (n=5 for all groups except n=7 for 1%+NAM).

	18%	1%	1%+NAM	1%+NR	1%+TRP	1%+MET
AMP	2.92±0.54	1.66±0.09	2.74±0.41	2.32±0.22	2.28±0.17	2.07±0.18
ADP	2.32±0.45	1.46±0.11	1.66±0.20	1.45±0.27	1.39±0.19	1.71±0.31
ATP	1.34±0.14	0.74±0.10	1.93±0.36	1.67±0.17	1.72±0.38	1.71±0.25
GMP	3.27±1.24	0.35±0.11	0.86±0.17	0.82±0.28	0.76±0.25	1.21±0.29
GDP	0.31±0.02	0.27±0.03	0.35±0.04	0.24±0.02	0.29±0.02	0.38±0.06
GTP	0.036±0.006	0.034±0.009	0.082±0.025	0.052±0.010	0.062±0.013	0.062±0.016
UMP	6.50±1.74	0.73±0.19	1.64±0.31	1.54±0.46	1.12±0.34	1.88±0.33
UDP	0.033±0.005	0.020±0.002	0.030±0.004	0.026±0.004	0.024±0.006	0.024±0.005
UTP	0.58±0.12	0.26±0.05	1.36±0.33	0.76±0.23	1.03±0.35	0.62±0.16
cyclic-ADP-ribose	1.22±0.27	0.53±0.11	1.25±0.33	1.23±0.12	0.97±0.19	1.27±0.25
cyclic-AMP	0.015±0.002	0.010±0.001	0.018±0.002	0.012±0.001	0.014±0.002	0.023±0.003
6-P-Gluconate	3.85±0.88	2.38±0.89	3.73±0.65	5.05±1.22	6.33±0.68	2.64±1.24
Acetylglucosamine-1P	17.21±1.78	5.96±0.79	8.24±1.05	6.66±0.59	8.61±1.16	6.77±0.50
Acetyl-Phosphate	12.01±1.277	15.37±4.28	13.88±2.46	13.44±2.29	17.75±1.17	6.98±1.11
ADP-Glucose*	4.476±0.992	1.450±0.590	4.617±1.316	3.134±1.325	2.830±0.602	1.488±0.244
2,3-bisP-Glycerate	0.048±0.019	0.033±0.003	0.121±0.038	0.034±0.014	0.088±0.027	0.078±0.032
DHAP	1.21±0.13	1.58±0.36	2.57±0.45	1.69±0.32	2.87±0.35	1.41±0.26
Total Fructose-bisP/Glucose-1,6-bisP	0.56±0.10	0.62±0.19	1.08±0.11	1.09±0.17	1.28±0.14	0.89±0.13
Fructose-6P	54.43±6.12	70.20±14.78	56.35±9.24	52.69±9.89	69.16±11.84	28.96±2.94
Glucosamine-6P	3.22±0.36	2.76±0.41	2.31±0.42	4.25±1.03	3.79±1.28	1.88±0.57
Glycerate-2/3-P	11.23±6.28	5.90±2.25	11.19±2.44	11.47±2.42	16.14±1.70	12.48±1.80
Glycerol-3-P	9.73±1.25	12.18±3.24	14.91±3.98	22.96±6.33	41.01±13.60	6.66±1.19
PEP	3.32±0.50	3.31±0.97	5.99±1.12	6.52±1.18	7.68±0.59	7.25±1.10
Phosphocreatine	0.021±0.012	0.010±0.003	0.020±0.006	0.010±0.003	0.022±0.009	0.018±0.010
Ribulose-1,5-bisP	0.16±0.05	0.07±0.01	0.20±0.05	0.15±0.03	0.17±0.02	0.10±0.03
Ribulose-5P	32.84±5.50	24.54±5.54	35.74±3.75	30.81±2.87	33.52±4.50	22.95±2.74
Sedoheptulose-7P	3.89±0.72	8.52±2.71	11.18±2.91	11.18±2.62	15.63±1.60	4.36±1.93
UDP-Glucose	0.138±0.041	0.013±0.003	0.560±0.171	0.167±0.148	0.192±0.111	0.063±0.040
Acetyl-CoA*	3.328±0.096	4.388±0.414	3.400±0.247	2.962±0.198	3.256±0.184	2.820±0.389
Hs-CoA	0.035±0.014	0.010±0.001	0.084±0.023	0.023±0.013	0.081±0.035	0.021±0.007
Malonyl-CoA*	4.556±0.953	9.842±1.136	4.426±0.497	2.760±0.354	3.648±0.529	2.952±0.976
Succinyl-CoA	0.209±0.125	0.023±0.004	0.145±0.049	0.059±0.021	0.099±0.030	0.090±0.052
Erythrose-4P	0.260±0.030	0.494±0.064	0.771±0.167	0.461±0.025	0.614±0.037	0.404±0.038
Glucose	4800±434	2611±628	2580±437	2775±658	3171±220	2211±358
Glucose-6P	23.13±5.99	55.89±15.89	52.61±8.96	36.22±11.84	54.96±13.60	6.90±1.46
Glycerylaldehyde-3P	2.20±0.15	5.38±0.74	8.90±1.62	5.82±1.01	7.30±1.15	5.55±0.60
Mannose-6P	14.17±0.68	34.35±11.16	27.54±3.07	21.03±6.66	34.70±13.02	11.55±4.05
Ribose-5P	9.81±1.19	10.93±1.72	16.05±2.00	14.71±3.07	14.48±1.99	8.79±2.39
α-Hydroxyglutaric acid	42.00±16.04	4.14±1.08	6.06±0.93	5.97±0.96	8.00±0.64	4.48±1.24
α-Ketoglutaric acid	9.36±3.43	3.57±1.14	3.30±1.40	3.56±1.35	2.57±0.57	4.21±0.96
Citric acid	0.58±0.17	33.21±12.72	34.55±14.69	14.55±14.13	44.46±20.43	21.74±10.54
Fumaric acid	985.0±108.3	1005.0±217.2	796.9±171.3	931.1±173.0	727.6±107.6	672.6±132.9
Glycolic acid	39.78±3.08	41.27±6.93	110.38±47.27	69.96±21.83	69.31±11.18	29.45±0.92
Isocitric acid	1.52±0.19	2.78±1.30	2.71±0.86	2.15±0.59	3.49±1.31	1.52±0.36
Latic acid	9340±1005	4437±1043	7080±1412	6103±1272	6603±918	5818±706
Malic acid	1236.8±29.2	1106.8±261.4	1311.7±303.8	1154.6±236.3	1397.8±161.1	994.7±189.3
Pyruvic acid	90.05±12.46	51.59±7.02	67.19±9.69	77.38±9.79	63.52±8.16	65.76±7.51
Succinic acid	21.62±5.00	13.30±5.56	17.30±10.61	5.37±1.14	9.44±1.01	5.00±1.03

Data are shown as the mean ± S.E.M. All units are presented as nmol/g, unless indicated (*) which are in nmol/kg.

Supplementary Table 2

Supplementary Table 2 Hepatic lipidomic profiles of total lipid for each class (n=6 per group).

		18%	1%	1%+NAM	1%+NR	1%+TRP	1%+MET
Totals for each class	Glycerides (Gly)	256.5±181.6*	558.3±235.7	597.2±84.9	485.5±248.4	529.4±78.7	724.0±94.6
	Phospholipids (PLs)	638.2±169.5**	268.0±175.7	249.5±85.1	200.4±135.7	244.6±77.3	156.5±63.1
	Sphingolipids (SLs)	26.1±5.5**	44.7±13.8	44.2±5.2	41.2±20.8	41.3±3.2	42.4±7.6
	Sterols	13.8±4.7**	111.0±64.7	81.3±21.6	89.3±56.0	167.8±71.8	64.3±39.9
Ratio	Gly/PLs	0.5±0.6	4.2±4.7	2.7±1.2	2.8±1.3	2.4±0.8	6.1±4.7
	Gly/SLs	9.1±4.0	12.2±2.9	13.5±0.9	11.8±1.0	12.8±1.1	17.2±1.0*
	Gly/Sterols	23.2±26.0	7.9±6.5	7.7±2.0	6.1±2.3	4.2±3.3	18.1±14.9

Gly: Glycerides. PLs: Phospholipids. SLs: Sphingolipids. $^{*}p < 0.05$, $^{**}p < 0.01$, $^{***}p < 0.001$, ns as not significant (versus the 1% group), one-way ANOVA followed by Tukey's post hoc test. Data are shown as the mean ± S.E.M.

Supplementary Table 3

Supplementary Table 3 List of antibodies used for western blot and immunofluorescent (related to Methods)

Antibody	Company	Product Number	Dilution	Species
SIRT1	Cell Signaling	2028	1:500	Rabbit
PGC-1α	Abcam	Ab54481	1:500	Rabbit
LC3B	Cell Signaling	2775	1:1000	Rabbit
β-actin	Abcam	Ab8227	1:1000	Mouse
Complex I	Abcam	Ab110242	1:1000	Rabbit
Complex IV	Santa Cruz	Sc13156	1:1000	Mouse
Complex V	Abcam	Ab14748	1:1000	Rabbit
4E-BP1	Cell Signaling	9644	1:1000	Rabbit
p-4E-BP1	Cell Signaling	2855	1:1000	Rabbit
TOM20	Santa Cruz	Sc11415	1:1000	Rabbit
HSP60	Abcam	Ab46798	1:1000	Rabbit
Ac-p53	Cell Signaling	2570	1:500	Rabbit
p53	Cell Signaling	2524	1:500	Mouse
DAPI	Abcam	Ab56788	1:1000	Rabbit
BODIPY	Invitrogen	D3922	1:1000	Rabbit
Anti-Rabbit	Invitrogen	026102	1:1000	
Anti-Mouse	Invitrogen	026502	1:1000	
Goat anti-rabbit IgG	Thermos Fisher Scientific	A11011	1:1000	

SIRT1: NAD-dependent deacetylase sirtuin-1. PGC-1α: the peroxisome proliferator-activated receptor-gamma coactivator-1alpha. LC3B: autophagy marker light chain 3B. β-actin: beta-actin (human gene and protein abbreviation ACTB/ACTB). 4E-BP1: Eukaryotic translation initiation factor 4E (eIF4E)-binding protein 1. p-4E-BP1: phosphorylated eukaryotic translation initiation factor 4E (eIF4E)-binding protein 1. TOM20: the mitochondrial preprotein translocases of the outer membrane 20. BODIPY: fluorinated boron-dipyrromethene. HSP60: heat shock protein 60. DAPI: 4',6-diamidino-2-phenylindole, dihydrochloride. Goat anti-rabbit IgG: goat anti-rabbit Immunoglobulin G.

Supplementary Table 4

Supplementary Table 4 List of primers used for qPCR (related to Methods)

Gene		Forward and Reverse primer (5' to 3')	Size
mtDNA	F	CCCAGCTACTACCATCATTCAAGT	117
	R	GATGGTTTGGGAGATTGGTTGATGT	
β-globin	F	AAGGTGAACGCCGATGAAGT	81
	R	ATCAAAGTACCGCTGGGTCC	
Acaa2	F	CCTGCTACGAGGTGTGTTCA	76
	R	GAAGTCCTTGAGAAGGCCCC	
Acadl	F	TGCACACATACAGACGGTGC	130
	R	CATGGAAGCAGAACCGGAGT	
Acadm	F	TGACAAAAGCGGGGAGTACC	144
	R	CCATACGCCAACTCTTCGGT	
Hadha	F	AGTGGAAAGCGTGACTCCAG	115
	R	GTAGTGCATGCCGATCACCT	
Acaca	F	CGATCTATCCGTCGGTGGTC	118
	R	GGTCTGCCATCTTAATGTATTCTGC	
Fasn	F	TGCACCTCACAGGCATCAAT	104
	R	GTCCCACTTGATGTGAGGGG	
Nnmt	F	GAGCCTTTGACTGGTCCCCA	95
	R	TGCACGCCTCAACTTCTCCT	
Gnmt	F	AACCGGAGGAAAGAGCCATCC	161
	R	TGCTCGCTCTGGTCACCTTTG	
Gamt	F	CCAGTGATGGAGCGTTGGGA	178
	R	GACGCTGGAAGACCCCATCA	
Pemt	F	CGGCAATATCGACTTCAGGCAG	99
	R	GTGATCACAGCCGCCACAAA	
Bhmt	F	CAGCCCCTGGCCTACCATAC	91
	R	TGGCAACTCGGGGTTCCAAT	
Rpl13a	F	TCCCTCCACCCTATGACAAG	136
	R	GTCACTGCCTGGTACTTCC	

mtDNA: mitochondrial DNA. *β-globin*: beta globin. *Acaa2*: acetyl-CoA acyltransferase 2. *Acadl*: acyl-CoA dehydrogenase long chain. *Acadm*: acyl-CoA dehydrogenase medium-chain. *Hadha*: hydroxyacyl-CoA dehydrogenase trifunctional multienzyme complex subunit alpha. *Acaca*: acetyl-CoA carboxylase alpha. *Fasn*: fatty acid synthase. *Nnmt*: nicotinamide N-methyltransferase. *Gnmt*: glycine N-methyltransferase. *Gamt*: guanidinoacetate N-methyltransferase. *Pemt*: phosphatidylethanolamine N-Methyltransferase. *Bhmt*: betaine-Homocysteine S-Methyltransferase. *Rpl13a*: ribosomal protein l13a (reference gene).

Supplementary Table 5

Supplementary Table 5 Diet component.

Ingredient	18% protein diet (g/kg) TD.180483	1% protein diet (g/kg) TD.180481
Casein	207.0	11.5
DL-Methionine	2.70	0.36
Sucrose	350	350
Corn Starch	256.1	431.2
Maltodextrin	50.0	50.0
Corn Oil	52.6	54.2
Cellulose	41.06	60.6
Ethoxyquin, antioxidant	0.01	0.01
Mineral Mix, Ca-P Deficient (79055)	13.37	13.37
Calcium Phosphate, dibasic	17.36	22.32
Calcium Carbonate	4.6	1.1
p-Aminobenzoic Acid	0.1101	0.1101
Vitamin C, ascorbic acid, coated (97.5%)	1.0166	1.0166
Biotin	0.0004	0.0004
Vitamin B12 (0.1% in mannitol)	0.0297	0.0297
Calcium Pantothenate	0.0661	0.0661
Choline Dihydrogen Citrate	3.497	3.497
Folic Acid	0.002	0.002
Inositol	0.1101	0.1101
Vitamin K3, menadione	0.0496	0.0496
Pyridoxine HCl	0.022	0.022
Riboflavin	0.022	0.022
Thiamin (81%)	0.022	0.022
Vitamin A Palmitate	0.0396	0.0396
Vitamin D3, cholecalciferol	0.0044	0.0044
Vitamin E, DL-alpha tocopheryl acetate	0.2423	0.2423
