# Supplementary material for: The role of the tryptophan-NAD + pathway in a mouse model of severe malnutrition induced liver dysfunction
Source: Nat Commun. 2022 Dec 8;13:7576. doi: 10.1038/s41467-022-35317-y (PMC9732354; doi:10.1038/s41467-022-35317-y)
Supplement: Supplementary file 2 — Description of Additional Supplementary Files [file 41467_2022_35317_MOESM2_ESM.pdf]

Description of Additional Supplementary Files

File Name: Supplementary Data 1
Description: List of lipids measured by Core Metabolomics and Lipidomics Laboratory, University of Cambridge, UK, and list of metabolites measured by The Metabolomics Innovation Centre, Canada.
