# Supplementary material for: The role of the tryptophan-NAD + pathway in a mouse model of severe malnutrition induced liver dysfunction
Source: Nat Commun. 2022 Dec 8;13:7576. doi: 10.1038/s41467-022-35317-y (PMC9732354; doi:10.1038/s41467-022-35317-y)
Supplement: Supplementary file 4 — Reporting Summary [file 41467_2022_35317_MOESM4_ESM.pdf]

nature portfolio

Corresponding author(s): Robert H. J. Bandsma
Last updated by author(s): Nov 7, 2022

Reporting Summary

Nature Portfolio wishes to improve the reproducibility of the work that we publish. This form provides structure for consistency and transparency in reporting. For further information on Nature Portfolio policies, see our Editorial Policies and the Editorial Policy Checklist.

Statistics

For all statistical analyses, confirm that the following items are present in the figure legend, table legend, main text, or Methods section.

n/a	Confirmed	
☐	☒	The exact sample size (*n*) for each experimental group/condition, given as a discrete number and unit of measurement
☐	☒	A statement on whether measurements were taken from distinct samples or whether the same sample was measured repeatedly
☐	☒	The statistical test(s) used AND whether they are one- or two-sided<br>*Only common tests should be described solely by name; describe more complex techniques in the Methods section.*
☒	☐	A description of all covariates tested
☒	☐	A description of any assumptions or corrections, such as tests of normality and adjustment for multiple comparisons
☐	☒	A full description of the statistical parameters including central tendency (e.g. means) or other basic estimates (e.g. regression coefficient) AND variation (e.g. standard deviation) or associated estimates of uncertainty (e.g. confidence intervals)
☐	☒	For null hypothesis testing, the test statistic (e.g. *F*, *t*, *r*) with confidence intervals, effect sizes, degrees of freedom and *P* value noted<br>*Give P values as exact values whenever suitable.*
☒	☐	For Bayesian analysis, information on the choice of priors and Markov chain Monte Carlo settings
☒	☐	For hierarchical and complex designs, identification of the appropriate level for tests and full reporting of outcomes
☒	☐	Estimates of effect sizes (e.g. Cohen's *d*, Pearson's *r*), indicating how they were calculated

Our web collection on statistics for biologists contains articles on many of the points above.

Software and code

Policy information about availability of computer code

Data collection	Panoramic Viewer v1.15 and Wave v2.6.1.53
Data analysis	R software v3.5.2, Prism GraphPad v9.0, ImageJ v1.52, Python v3.7.2 and MetaboAnalyst v4.0,

For manuscripts utilizing custom algorithms or software that are central to the research but not yet described in published literature, software must be made available to editors and reviewers. We strongly encourage code deposition in a community repository (e.g. GitHub). See the Nature Portfolio guidelines for submitting code & software for further information.

Data

Policy information about availability of data

All manuscripts must include a data availability statement. This statement should provide the following information, where applicable:

- Accession codes, unique identifiers, or web links for publicly available datasets
- A description of any restrictions on data availability
- For clinical datasets or third party data, please ensure that the statement adheres to our policy

All relevant data of this study are available within the paper and its supplementary information files. Source data are provided as a Source Data file. All data that support this study are available from the corresponding authors upon reasonable request.

Human research participants

Policy information about studies involving human research participants and Sex and Gender in Research.

Reporting on sex and gender	N/A
Population characteristics	N/A
Recruitment	N/A
Ethics oversight	N/A

Note that full information on the approval of the study protocol must also be provided in the manuscript.

Field-specific reporting

Please select the one below that is the best fit for your research. If you are not sure, read the appropriate sections before making your selection.

☒ Life sciences ☐ Behavioural & social sciences ☐ Ecological, evolutionary & environmental sciences

For a reference copy of the document with all sections, see nature.com/documents/nr-reporting-summary-flat.pdf

Life sciences study design

All studies must disclose on these points even when the disclosure is negative.

Sample size	Sample size on animal studies were determined based on a power of 80% (5% two-sided) and the standard deviations and noise ratios generally observed in initial pilot studies. The sample size of experiment measurements involving qPCR, western blot and immunostaining follows common standards employing three or more biological replicates, which is based on extensive laboratory experience and literature in the field (PMID: 19570514, 23261783, 32130914 and 32076644, n = 3 was typically minimum standards in the field). Sample size is reported in the legends for all figures.
Data exclusions	No data were excluded from the analysis.
Replication	Reproducibility was assessed by replicas and repeated experiments. Results shown in the manuscript are representative of at least two times and got similar results.
Randomization	Animals were distributed in a random fashion between experimental groups.
Blinding	The investigators were blinded to group allocation and metabolomic analyses. Image acquisition and quantification were also performed in a blinded fashion.

Reporting for specific materials, systems and methods

We require information from authors about some types of materials, experimental systems and methods used in many studies. Here, indicate whether each material, system or method listed is relevant to your study. If you are not sure if a list item applies to your research, read the appropriate section before selecting a response.

Materials & experimental systems

n/a	Involved in the study	
☐	☒	Antibodies
☒	☐	Eukaryotic cell lines
☒	☐	Palaeontology and archaeology
☐	☒	Animals and other organisms
☒	☐	Clinical data
☒	☐	Dual use research of concern

Methods

n/a	Involved in the study	
☒	☐	ChIP-seq
☒	☐	Flow cytometry
☒	☐	MRI-based neuroimaging

Antibodies

Antibodies used	All antibodies used with catalog numbers and dilution are specified in Supplementary Table 3.

Validation	All antibodies were commercial and validated for the given species and application, either directly by the manufacturers or in the primary references cited and visible on the websites of the products:<br>SIRT1 Cell Signaling 20285 https://www.cellsignal.com/products/primary-antibodies/sirt1-antibody-mouse-specific/2028<br>PGC-1α Abcam Ab54481 https://www.abcam.com/pgc1-alpha--beta-antibody-bsa-and-azide-free-ab54481.html<br>LC3B Cell Signaling 2775 https://www.cellsignal.com/products/primary-antibodies/lc3b-antibody/2775<br>β-actin Abcam Ab8227 https://www.abcam.com/beta-actin-antibody-ab8227.html<br>Complex I Abcam Ab110242 https://www.abcam.com/ndufb8-antibody-20e9dh10c12-ab110242.html<br>Complex IV Santa Cruz Sc13156 https://www.scbt.com/p/cytochrome-c-antibody-a-8<br>Complex V Abcam Ab14748 https://www.abcam.com/atp5a-antibody-15h4c4-mitochondrial-marker-ab14748.html<br>4E-BP1 Cell Signaling 9644 https://www.cellsignal.com/products/primary-antibodies/4e-bp1-53h11-rabbit-mab/9644<br>p-4E-BP1 Cell Signaling 2855 https://www.cellsignal.com/products/primary-antibodies/phospho-4e-bp1-thr37-46-236b4-rabbit-mab/2855<br>TOM20 Santa Cruz Sc11415 https://www.scbt.com/p/tom20-antibody-fl-145<br>HSP60 Abcam Ab46798 https://www.abcam.com/hsp60-antibody-ab46798.html<br>Ac-p53 Cell Signaling 2570 https://www.cellsignal.com/products/primary-antibodies/acetyl-p53-lys379-antibody/2570<br>p53 Cell Signaling 2524 https://www.cellsignal.com/products/primary-antibodies/p53-1c12-mouse-mab/2524<br>DAPI Abcam Ab56788 https://www.abcam.com/drp1-antibody-3b5-ab56788.html<br>BODIPY Invitrogen D3922 https://www.thermofisher.com/order/catalog/product/D3922<br>Anti-Rabbit Invitrogen 026102 https://www.thermofisher.com/antibody/product/Rabbit-IgG-Isotype-Control/02-6102<br>Anti-Mouse Invitrogen 026502 https://www.thermofisher.com/antibody/product/Mouse-IgG-Isotype-Control/02-6502<br>Goat anti-rabbit IgG Thermos Fisher Scientific A11011 https://www.thermofisher.com/antibody/product/Goat-anti-Rabbit-IgG-H-L-Cross-Adsorbed-Secondary-Antibody-Polyclonal/A-11011

Animals and other research organisms

Policy information about studies involving animals; ARRIVE guidelines recommended for reporting animal research, and Sex and Gender in Research

Laboratory animals	Mice (C57BL/6 male), 3 weeks postpartum, temperature and humidity (23°C, 40%-60%), housed socially (3-5 per cage).
Wild animals	No wild animals were used in the study.
Reporting on sex	Male
Field-collected samples	No field collected samples were used in the study.
Ethics oversight	All animal experiments were approved by the Animal Care Committee of The Hospital for Sick Children, Toronto (Animal Use Protocol Number: 1000030900).

Note that full information on the approval of the study protocol must also be provided in the manuscript.
